# Investigation into the Potential Mechanism of Radix Paeoniae Rubra Against Ischemic Stroke Based on Network Pharmacology

**DOI:** 10.3390/nu16244409

**Published:** 2024-12-23

**Authors:** Tingyu Wen, Guang Xin, Qilong Zhou, Tao Wang, Xiuxian Yu, Yanceng Li, Shiyi Li, Ying Zhang, Kun Zhang, Ting Liu, Beiwei Zhu, Wen Huang

**Affiliations:** 1 Department of Emergency Medicine, Natural and Biomimetic Medicine Research Center, Tissue-Orientated Property of Chinese Medicine Key Laboratory of Sichuan Province, West China School of Medicine, West China Hospital, Sichuan University, Chengdu 610000, China; wtyaimm@163.com (T.W.); xinguang@scu.edu.cn (G.X.); qlzhou@scu.edu.cn (Q.Z.); terrywang1126@scu.edu.cn (T.W.); yuxiuxian@wchscu.cn (X.Y.); lycyancen@163.com (Y.L.); lsyshiyi@163.com (S.L.); zhang_ying@stu.scu.edu.cn (Y.Z.); zhangkun98163@163.com (K.Z.); liuting526@wchscu.cn (T.L.); 2 State Key Laboratory of Marine Food Processing and Safety Control, National Engineering Research Center of Seafood, Dalian Polytechnic University, Dalian 116034, China

**Keywords:** ischemic stroke, Radix Paeoniae Rubra, platelet, albiflorin, β-sitosterol

## Abstract

Background: Radix Paeoniae Rubra (RPR), an edible and medicinal Traditional Chinese Medicine (TCM), is extensively employed in therapeutic interventions of cardiovascular and cerebrovascular diseases. However, the curative effect of RPR on ischemic stroke remains ambiguous. This work integrated network pharmacology, molecular docking, and experimental validation to explore the mechanisms of RPR in treating ischemic stroke. Methods: In this study, we preliminarily elucidated the therapeutic effect and mechanism of RPR on ischemic stroke through network pharmacology, molecular docking analysis, and experimental verification. Results: The results indicated that RPR improved the neurological deficit scores, decreased the size of infarcts, and reduced brain edema symptoms in the tMCAO mice model. Furthermore, through network pharmacology and molecular docking, four core targets (MAPK3, TNF-α, MAPK14, and JNK) closely related to RPR’s treatment of ischemic stroke were identified, exhibiting strong affinity with two key active components of RPR: albiflorin (AF) and β-sitosterol (BSS). The Western blot showed the potential mechanism of RPR treatment for ischemic stroke by regulating the MAPK signaling pathway. Moreover, RPR and its main active ingredients exhibited a significant inhibitory effect on platelets. Conclusion: In conclusion, this study revealed that RPR alleviates ischemic injury by activating the MAPK signaling pathway, and its protective effect may partly stem from inhibiting platelet activation. This work may provide a scientific basis for the development and utilization of RPR as a natural edible material to prevent ischemic stroke and anti-platelet therapy.

## 1. Introduction

Stroke is an acute cerebrovascular disease whose core etiology lies in the sudden rupture or occlusion of cerebral blood vessels, a process that directly leads to cerebral ischemia/hypoxia injury [1]. Stroke falls into two distinct categories: hemorrhagic and ischemic strokes [2]. Ischemic stroke comprises a substantial portion, estimated to be roughly 76%, of the total strokes [3]. The primary treatment strategy for acute ischemic stroke involves the rapid restoring of blood flow through thrombolytic therapy or mechanical thrombectomy. However, even after a successful reperfusion of blood flow, the expansion of the infarcted region may persist, a condition designated as cerebral ischemia/reperfusion injury (CI/RI) [4]. Studies indicated that the abnormal activation of platelets plays a significant role in this pathological process. Activated platelets facilitate the formation of blood clots in circulation, which, upon blocking cerebral arteries, can directly trigger a stroke [5]. Platelet activation is accompanied by the transduction of intracellular signaling involving the rearrangement of the cytoskeletal system, the release of α- and dense granules, and the conformational change in the affinity state of integrin adhesion receptors (particularly αIIbβ3). These biological processes intertwine and collectively contribute to the progression of the disease [4,5]. Therefore, this study aims to elucidate the protective role and mechanism of RPR in ischemic stroke by influencing platelet activation.

With the continuous development of Traditional Chinese Medicine (TCM), research on the treatment of cardiovascular and cerebrovascular diseases using TCM is gradually increasing. In traditional Chinese medicine theory, ischemic stroke is regarded as a disease caused by qi stagnation and blood stasis [6]. Consequently, medications with the functions of stimulating the circulation of blood and eliminating stagnation are employed to treat ischemic stroke [1]. These medications alleviate and treat ischemic stroke by enhancing blood circulation, eliminating blood clots, and improving cerebral blood supply.

Radix Paeoniae Rubra (RPR), the dried root of either Paeonia lactiflora Pall. or Paeonia veitchii Lynch, exhibits the effects of clearing blood heat, ameliorating blood stasis, and alleviating pain [7]. In TCM clinical practice, RPR is commonly used to treat warm-toxin diseases, accompanied by ecchymoses, nosebleeds, swollen and painful eyes, hypochondriac pain, abdominal pain, amenorrhea, dysmenorrhea, bloody diarrhea, and to disperse swelling [8]. Studies have demonstrated that RPR and its extracts exhibit various activities, including anti-inflammatory, neuroprotective, cardioprotective, hepatoprotective, antidiabetic, anti-gastric ulcer, anti-allergic, and antiviral effects [8]. Nevertheless, the therapeutic effects and molecular mechanisms of RPR on ischemic stroke remain incompletely understood.

Network pharmacology has garnered extensive usage in pinpointing potential bioactive ingredients and forecasting disease targets within Traditional Chinese Medicine (TCM) [9]. By integrating vast network database resources, along with the integration of chemical informatics and bioinformatic methodologies, network pharmacology constructs an all-inclusive framework that integrates drugs with their corresponding disease targets, forming a comprehensive network. This network can be leveraged to predict the potential active ingredients, target effects, and mechanisms of action of TCM, thereby facilitating the overcoming of challenges posed by the “multi-component, multi-target” of TCM [3].

In the present study, we delved into the therapeutic effects and molecular mechanisms of RPR and its main components in treating ischemic stroke using a combined approach of network pharmacology, molecular docking, and experiment validation. Our results demonstrated that RPR could reduce brain damage induced by tMCAO via the MAPK pathway. Furthermore, by employing the combined techniques of network pharmacology and molecular docking, we confirmed that two main ingredients in RPR, albiflorin (AF) and β-sitosterol (BSS), exhibit antiplatelet activity. These results indicated that the therapeutic effects of RPR on ischemic stroke may be partially due to its antiplatelet activation capabilities.

## 2. Materials and Methods

### 2.1. Reagents and Materials

RPR was obtained from Beijing Tongrentang Co., Ltd. (Beijing, China). The RPR was made into decoction, and the decoction was made into fine particles by spray-drying technology. Before administration, the RPR powder was made into 1 × PBS buffer solution of different concentrations according to the administered dose, added to warmth, and stirred well.

Albiflorin (A138782) and β-sitosterol (S434260) were obtained from Shanghai Aladdin Biochemical Technology Co., Ltd. (Shanghai, China). A cell counting kit-8 (C0037) and CellTiter-LumiTM cell viability assay kit (C0069S) was purchased from Beyotime Biotechnology (Shanghai, China). The PF4 ELISA kit was obtained from Shanghai Weiao Biotechnology Co., Ltd. (Shanghai, China). FluorTM 488 phalloidins (40736ES75) were acquired from Yeasen Biotechnology Co., Ltd. (Shanghai, China). Additionally, goat anti-rabbit IgG-HRP (SA00001-2) and goat anti-mouse IgG-HRP (SA00001-1) were bought from Proteintech (Wuhan, China). ZO-1 (sc-33725), Occludin (sc-133256) and Claudin-5 (sc-374221) were acquired from Santa Cruz Biotechnology (Santa Cruz, CA, USA). p38(WL00764), p-p38(WL03428), JNK(WL01295), and P-JNK(WL01813) antibodies were acquired from Shenyang Wanlei Biotechnology Co., Ltd. (Shenyang, China). ERK (4695) and p-ERK(4370) were acquired from Cell Signaling Technology, Inc. (Boston, MA, USA). IL-6 and IL-1β ELISA kits were acquired from Shanghai Enzymes Biotechnology Co., Ltd. (Shanghai, China).

### 2.2. Target Collection and Network Construction

Traditional Chinese Medicine Systems Pharmacology (TCMSP) was used to obtain the main ingredients in RPR, and 13 main active ingredients were filtered out based on oral efficacy (OB) > 30% and drug similarity (DL) > 0.18. The targets associated with these main ingredients were identified through the SwissTargetPrediction (http://www.swisstargetprediction.ch/ accessed on 10 May 2024) and PharmcMapper Server (https://www.lilab-ecust.cn/pharmmapper/ accessed on 10 May 2024) databases. Additionally, the stroke-related targets were obtained from GeneCards (https://www.genecards.org/ accessed on 11 May 2024), DisGeNET (https://www.disgenet.org/ accessed on 11 May 2024), and OMIM (https://omim.org/ accessed on 11 May 2024) databases, respectively.

The identification of 128 common targets intersected by RPR and stroke were achieved utilizing Venny 2.1.0. For network visualization, Cytoscape version 3.9.0 was employed. Based on the results, a node’s significance was directly proportional to its score, visually represented by a darker hue.

### 2.3. Protein–Protein Interaction (PPI) and Enrichment Analyses

The PPI (protein–protein interaction) network encompassing 128 shared targets was constructed utilizing the STRING 11.0 database [10]. The minimum confidence level for the links was established at 0.7 or above. Within each sub-cluster, we identified the node with the highest score, labeled as SEEDs, indicating its potential to serve as the central target for that particular cluster. The “RPR-Component-Target” network and “Disease-Pathway-Target-Component-Drug” network were constructed using Cytoscape version 3.9.0. We used the DAVID V6.8 database to conduct a gene ontology (GO) enrichment analysis and a KEGG pathway analysis for the potential targets and signaling pathways of RPR in the treatment of ischemic stroke. The OmicShare platform was utilized to visually display the results.

### 2.4. Molecular Docking

We retrieved the structural information for compounds from PubChem and that for targets from the RCSB Protein Data Bank (PDB), respectively. Adhering to the guidelines provided in the AutoDock Vina tutorial and manual (accessible at http://ccsb.scripps.edu accessed on 10 May 2024), we employed AutoDock Tools-1.5.6 to prepare the ligands and receptors. Additionally, we leveraged PyMol to visualize the outcomes generated by AutoDock. Vina and PLIP. The Surflex-Dock (SFXC) mode of docking offers estimates of binding affinities, leveraging the SFXC scoring system to determine the strength of interactions [11].

### 2.5. HPLC Analysis of Primary Active Ingredients in RPR

High-Performance Liquid Chromatography (HPLC) systems were utilized to determine the primary active compounds of RPR. A precise measurement of 1.0 g of RPR extract was taken and subsequently dissolved in a 10 mL solution of 50% methanol. To prepare AF and BSS at a concentration of 0.1 mg/mL, methanol was employed as the solvent. Following this, the solution underwent filtration through a 0.22-micrometer filter. After being filtered, the aforementioned specimens were individually subjected to analysis using High-Performance Liquid Chromatography (HPLC) systems. For the identification of AF and BSS, the Shimadzu LC-2040C HPLC system, integrated with an evaporative light scattering detector (ELSD), was chosen. The ShimNex HE C18-AQ column (4.6 × 250 mm, 5 μm) was employed for the separation process. The ELSD operational parameters were set as follows: the drift tube temperature was adjusted to 50 °C, the gain value was 5, and the carrier gas pressure was maintained at 405 bar. The temperature of the column was maintained at 30 °C. A concentration of 0.1% formic acid–water served as mobile phase A, and acetonitrile served as mobile phase B. The elution ratio was A: B = 95: 5 for 5.00 min, A: B = 10: 90 for 15.00 min, A: B = 10: 90 for 23.00 min, and A: B = 83: 17 for 23.01 min. A volume of 10 μL was utilized for the analysis, with a flow rate set at 1 mL/min.

### 2.6. Animal Experiments

Male C57BL/6 mice (20–22 g, 6–7 weeks) were obtained from the Beijing Vital River Laboratory Animal Technology Company (Beijing, China). The mice were housed under standardized conditions, including a temperature range of 24 ± 2 degrees Celsius, a relative humidity of 50 ± 5%, and a 12 h light/12 h dark cycle. During an initial week of acclimation, they had unrestricted access to standard laboratory food and water. During the adaptive feeding period (one week), mice with a body weight of 20–22g and normal physiological performance were included in the experimental analysis. Other mice were excluded from experimental analysis. Throughout the course of the research, all experiments pertaining to animals were performed strictly to ethical guidelines and principles and guidelines outlined by the West China Hospital of Sichuan University for animal experimentation (Ethical number: 20220222071).

The mice were randomly assigned to four groups: a sham group, a tMCAO model group, and two RPR groups receiving low and high doses, respectively. The RPR extract was dissolved in 1 × PBS. The low-dose and high-dose groups were administered the substance orally at dosages of 50 mg/kg and 100 mg/kg, twice per day for three days in succession. Meanwhile, the sham and tMCAO groups were administered an equivalent volume of 1×PBS. Three days post-intervention, the tMCAO model was established via the implementation of a middle cerebral artery occlusion (MCAO) induction protocol [12]. Briefly, after anesthetization, a median incision was performed on the neck of each mouse to reveal the right common carotid artery (CCA), external carotid artery (ECA), and internal carotid artery (ICA). The EECA and CCA were ligated was ligated. Subsequently, a minute incision was made on the CCA, and a silicone-coated nylon monofilament was introduced into the middle cerebral artery (MCA) via the internal carotid artery (ICA). Following 1 h of ischemia, the monofilament was gradually withdrawn. For the sham-operated rats, the same surgical steps were followed as the experimental rats, except that no monofilament was introduced into the artery. The tMCAO induction procedures were performed in the morning to minimize the impact of circadian rhythm changes on outcome measures. After 24 h of reperfusion, the euthanasia of mice by intravenous injection of excessive pentobarbital anesthesia and a subsequent pharmacodynamic evaluation were conducted.

The mice showed that an unsteady gait or lameness or lack of responsiveness to manual stimuli or rough hair coat, and a lack of grooming were the humane endpoints established for this study. No adverse events were found in animal experiments.

### 2.7. TTC Staining

At 24 h post-reperfusion, the mice were euthanized while under anesthesia. Subsequently, the brain tissue was divided into four equal pieces with 2 mm slices. Following this, the cerebral tissue slices were immersed in a 2% TTC solution and maintained in the darkness at 37 °C for a duration of 30 min [13].

After capturing images of the stained brain slices, the cerebral infarct area was precisely measured and quantified through the utilization of the ImageJ image analysis software (ImageJ 1.42, Wayne Rasband, National Institutes of Health, Bethesda, MD, USA). The extent of cerebral ischemia/reperfusion injury (CI/RI) was quantitatively assessed by determining the infarct volume ratio, which was calculated as a percentage (%) by dividing the total cerebral infarct volume by the total brain volume and multiplying by 100.

### 2.8. Neurologic Deficit Score

After 24 h of reperfusion, the degree of neurological impairment was assessed using the neurologic deficit score. The neurological deficit score was assigned as follows: 0 for no observable neurological deficits, 1 for the inability to fully extend the left forepaw, 2 for the difficulty in extending the left forelimb and circling towards the left, 3 for the inability to move towards the left, and 4 for the absence of spontaneous walking or a diminished level of consciousness [14].

### 2.9. ELISA Analysis

After 24 h of reperfusion, the brain tissue was obtained after euthanizing the mice and prepared as tissue homogenate. IL-6 and IL-1β levels were assayed using a commercially available ELISA kit according to the protocol of manufacture.

### 2.10. Pathological Staining

After 24 h of reperfusion, brain tissues were preserved in a formaldehyde solution for fixation. Subsequently, the brain tissues embedded in paraffin were precisely sliced into sections of 50 μm in thickness and subjected to either hematoxylin and eosin (H&E) staining or Nissl staining for further analysis. Images were captured using a scanner (PerkinElmer, Waltham, MA, USA) at a magnification of 50× to obtain a clear representation.

### 2.11. Cell Viability Assay

After treatment with RPR, AF (dissolved with 1 × PBS) and BSS (dissolved with 1 × PBS) platelets were seeded in 96-well culture plates for 1 h. Subsequently, the cells were exposed to 10 μL of CCK8 and incubated at 37 °C for 1 h. Following this, the level of absorbance at the wavelength of 450 was quantified utilizing a microplate reader.

### 2.12. Western Blotting

The previously established protocol was adhered to for both the extraction of total protein and the subsequent measurement of protein concentration. Following denaturation, the sample was resolved using SDS-PAGE and subsequently transferred onto a PVDF membrane. The membrane was blocked with 5% bovine serum albumin (BSA) for 2 h at room temperature after the transmembrane step. Subsequently, the membranes underwent a 16 h incubation process at 4 °C with the primary antibodies. Subsequent to the incubation with the primary antibodies, excess antibodies were removed through a 30 min wash at room temperature utilizing TBST. HRP-conjugated rabbit or mouse IgG secondary antibodies were introduced and allowed to incubate for 2 h at room temperature. The density of the band was quantitatively determined using the ImageJ software application.

### 2.13. Platelet Preparation

Under pentobarbital anesthesia, blood samples were obtained from the hearts of mice and subsequently mixed with a 3.8% citrate-based anticoagulant. Blood samples were centrifuged at 250× *g* for 6 min to separate platelet-rich plasma (PRP), which was then further centrifuged at 800× *g* for 10 min to obtain the platelet. Platelets were gently resuspended in a HEPES/Tyrode (H-T) buffer (136 mM of NaCl, 0.4 mM of Na_2_HPO_4_, 2.7 mM of KCl, 12 mM of NaHCO_3_, 0.1% glucose, 0.35% BSA, pH 7.4) [15].

### 2.14. ATP Secretion Assay

After a 1 h treatment with RPR, AF and BSS platelets were centrifuged at 800× *g* for 10 min. A 100 μL supernatant was co-incubated with 100 μL of a Cell-Titer-Lumi^TM^ Steady luminescent solution at 37 °C for 10 min. The luminescence was then measured by a microplate reader [16].

### 2.15. Platelet Aggregation

After AF and BSS were treated with RPR for 1 h at 37 °C, the PRP was used to detect platelet aggregation. The platelet aggregation rate was quantified at 37 °C using the turbidimetric approach, which involved the utilization of the PAP-4 aggregator. The ultimate concentration of the inducer was set as follows: ADP, 1 μM and 2 μM; thrombin (Thr), 0.01 U/mL and 0.02 U/mL [16].

### 2.16. Platelet Clot Retraction

A 200 μL PRP concentration was mixed with an 800 μL H-T buffer and 5 μL of erythrocytes after being treated with RPR (AF and BSS) for 1 h at 37 °C. To initiate clot retraction, thrombin (1 U/mL) was introduced and allowed to react at room temperature for a duration of 45 min and recorded every 15 min.

### 2.17. Platelet Spreading on Coated Fibronectin

Washed platelets were treated with a concentration of 0.01 U/mL of thrombin and subsequently distributed onto well plates featuring a glass bottom, which had been coated with fibrinogen at a density of 100 μg/mL, where they were incubated for 45 min. After washing with a modified H-T buffer solution, the remaining adhered platelets were stabilized with 2% formaldehyde.

The cells underwent labeling with rhodamine-linked phalloidin and were subsequently observed and analyzed using a multiparametric cell dynamic analysis system (PE/Opera Phenix Plus). The ImageJ software, provided by NIH in Bethesda, MD, USA, was employed to analyze the images and quantify the extent of surface coverage of platelets adhering to fibrinogen [15].

### 2.18. Statistical Analysis

The data were presented as mean ± SD and were statistically analyzed by Graphpad Prism 9.0 (GraphPadSoftware Inc., San Diego, CA, USA). The Shapiro–Wilk test was administered to assess the normality of all dates. If normality was confirmed and there were no significant differences in variance between groups (F test), an unpaired Student’s t-test was used to analyze the statistical significance between two groups, and a one-way ANOVA with Dunnet’s post-test analysis was used to analyze the statistical significance among multiple groups. Where needed, a two-way ANOVA analysis was conducted, with Sidak’s correction applied for multiple comparisons. If data were not normally distributed, either a two-tailed Mann–Whitney U-test or a Kruskal–Wallis test with Dunn’s multiple comparison adjustment was carried out as specified. Since all data were found to be normally distributed, we did not exclude any data. No randomization or blinding was used in this study. *p* < 0.05 was considered statistically significantly different. The G*Power (version 3.1.9,2) for all the data presented in this study is greater than 0.8, indicating that the robustness and reliability of our findings and the sample size used in this study is reasonable.

## 3. Results

### 3.1. The Network Pharmacology Analysis of the RPR Targets Against Ischemic Stroke

**Figure 1:**
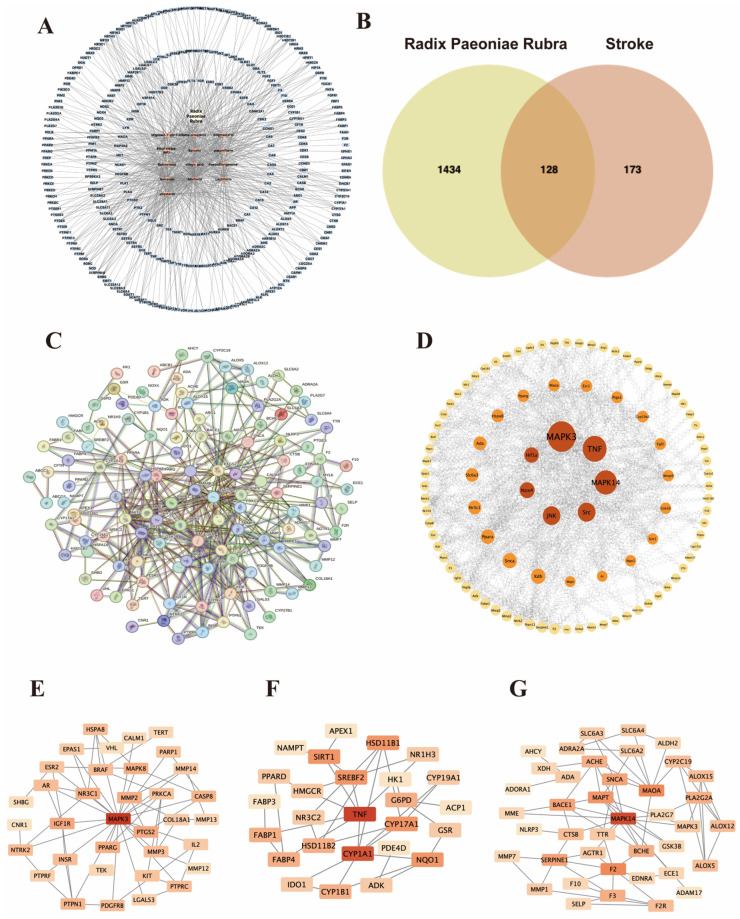
The network pharmacology analysis of RPR against stroke: (**A**) “RPR-Component-Target” network. The pink nodes represent the ingredients of RPR, and the blue nodes represent the targets, (**B**) The Venn diagram of 128 targets intersected by RPR and stroke. (**C**) The PPI network of the 128 common targets. (**D**) The core targets of the 128 common targets ranked by degree value. The node size and degree value are positively correlated. (**E**–**G**) A cluster analysis identifies the top 3 core seed nodes of the core intersection targets.

To find the potential therapeutic targets of RPR in protecting ischemic stroke, we initially applied a network pharmacology analysis to identify the potential targets of RPR. A total of 1562 specific targets were obtained from 13 active ingredients of RPR, which were collected from the TCMSP databases. (Figure 1A, Supplementary Table S1). A total of 356 ischemic stroke-related targets were obtained from GeneCards (scores > 15) (Supplementary Table S2), 16 ischemic stroke-related targets were obtained from OMIM (Supplementary Table S3), and 30 ischemic stroke-related targets were obtained from DisGeNET (Supplementary Table S4). Upon consolidating the findings from the aforementioned three databases, a total of 301 targets associated with ischemic stroke were identified (Supplementary Table S5). Upon intersecting the ischemic stroke-associated targets with the predicted RPR targets, we refined 128 common targets, which were subsequently identified as the pivotal targets for the therapeutic potential of RPR in treating stroke (Figure 1B).

The PPIs of overlapping 128 genes were constructed by the STRING and STITCH database. At a high confidence score (>0.70), a network was formed with 128 nodes, 435 edges, indicating that these genes have the highest likelihood of interaction (Figure 1C, Supplementary Figure S1). The PPI network generated from STRING was exported to Cytoscape version 3.9.0 for visualization and further analysis. According to degree centrality (DC) values, we identified the top 10 targets including MAPK3 (ERK1), TNF, MAPK14 (p38), SRC, etc. (Figure 1D, Table 1). Further examination of PPI network targets was conducted using MCODE, and three highly interactive clusters were identified (Figure 1E–G). The detailed characteristics pertaining to these clusters are presented in Table 2. Cluster 1 contains 48 targets with 48 nodes and 144 edges. The initiating node of cluster 1 is TNF (Figure 1E), which is a pivotal mediator in regulating immune responses involved in infection control, autoimmune conditions, allergic diseases, and antitumor activity. TNF-α triggers local inflammation and thrombosis by affecting the endothelium of blood vessels. Research has demonstrated that the interactions between TNF-α ligands and their receptors play a crucial role in various aspects of stroke-induced brain damage. Cluster 2 contains 46 targets with 46 nodes and 84 edges. The seed node of cluster 1 is MAPK3 (Figure 1F), one of serine/threonine protein kinases, and its pivotal significance has been shown in the regulation of cell mitogenesis, differentiation, synaptic transmission, and neuro-protective mechanisms. Cluster 3 contains 34 targets with 34 nodes and 41 edges. The seed node of cluster 1 is MAPK14 (Figure 1G), one of the serine/threonine protein kinases that is involved in the process of immune and inflammatory reactions. Previous research has suggested that p38 MAPK is involved in the inflammatory response of cerebral ischemia.

**Figure 2:**
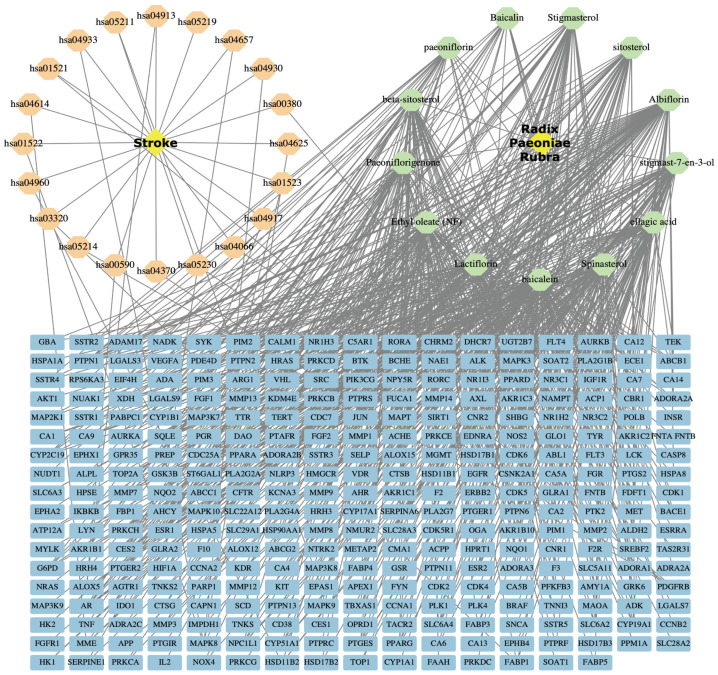
“Disease-Pathway-Target-Component-Drug” network. The orange nodes represent signaling pathway involved in stroke, the green nodes represent ingredients of RPR, and the blue nodes represent the common targets between stroke and RPR.

The visualization network diagram of “Stroke-Pathway-Target-Component-RPR” involves 13 active ingredients and 128 prospective therapeutic targets of RPR for ischemic stroke treatment, and the top 20 *p*-value pathways of ischemic stroke were obtained by Cytoscape version 3.9.0 (Figure 2). The outcomes conclusively exhibited the therapeutic potential of RPR in ischemic stroke through its multifaceted actions on multiple targets and pathways.

### 3.2. GO and KEGG Enrichment Analysis

**Figure 3:**
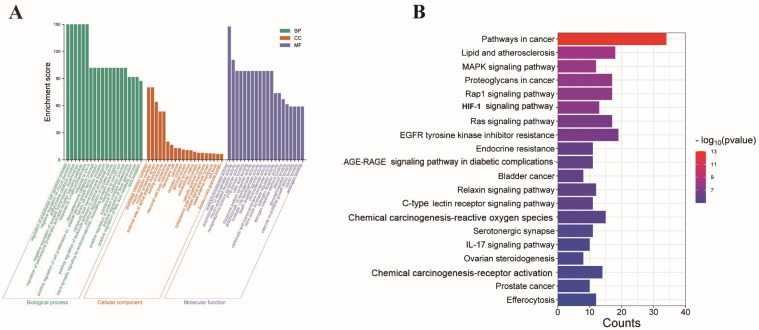
The enrichment analysis of 128 intersected targets: (**A**) the top 20 GO enrichment terms (BP: biological process, CC: cellular component, and MF: molecular function); (**B**) Top 20 KEGG enrichment analysis item.

Through enrichment analysis utilizing GO (Gene Ontology) enrichment, we have derived the top 20 most relevant biological processes (BPs), cellular components (CCs), and molecular functions (MFs) (Figure 3A). Among these items, biological processes (BPs) involve regulation of the endothelial cell apoptotic process and the negative regulation of mucus secretion. Cellular components (CCs) involve the peptidase inhibitor complex and insulin receptor complex. Molecular function (MF) involves acetylcholinesterase activity and arachidonate 12-lipoxygenase activity. According to the analysis of KEGG items, signaling pathways such as lipid and atherosclerosis, the MAPK signaling pathway, the Rap1 signaling pathway, and the HIF-1 signaling pathway were mainly involved in ischemic stroke response (Figure 3B).

### 3.3. Therapeutic Effects of RPR on Ischemic Stroke in tMCAO Mice

**Figure 4:**
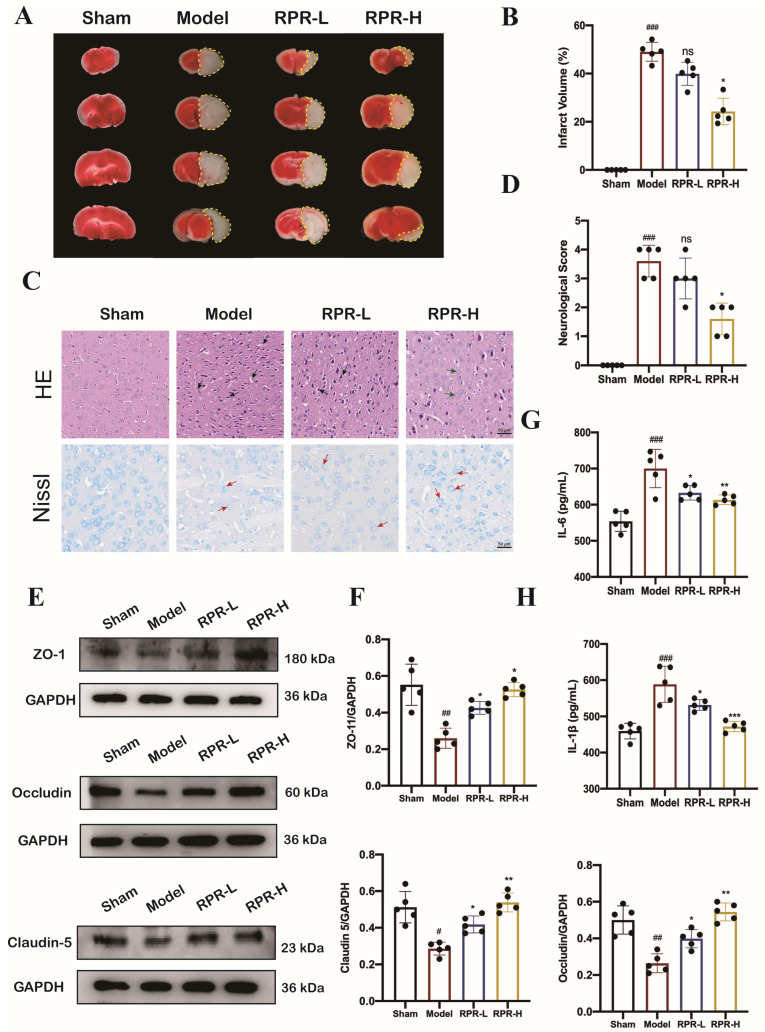
The protective effect of RPR on tMCAO mice: (**A**) Representative images of TTC staining of mice brain tissue with different dosages of RPR for 3 d of preventive administration, cerebral infarction area circled by yellow dashed, *n* = 5; (**B**) The quantification results of brain infarction volume; (**C**) H&E staining and Nissl staining images of brain tissues, *n* = 5. Scale bar = 50 μm. (**D**) Neurological score treated with different dosages of RPR, *n* = 5; (**E**,**F**) Representative Western blots showing ZO-1, Occludin, and Claudin- 5 levels, *n* = 5; (**G**,**H**) IL-6 level and IL-1β level in brain tissues (*n* = 5). The data are presented as mean ± SD. *# p* < 0.05, *## p* < 0.01, *### p* < 0.001 vs. Sham group, * p < 0.05, ** p < 0.01, *** p < 0.001 vs. Model group, ns, no significance.

The middle cerebral artery (MCA) is frequently the primary blood vessel affected in cases of ischemic stroke [4]. To delve into the fundamental pathomechanisms underlying ischemic stroke, the transient middle cerebral artery occlusion (tMCAO) model, which simulates focal cerebral ischemia, has been employed in this investigation. The protective effects of RPR on reducing infarction size were investigated in tMCAO mice by using TTC staining. As shown in Figure 4A,B, the ischemic-induced injury in the tMCAO group elicited a prominent elevation in infarct volume when compared to the sham group. The administration of high-dose RPR (RPR-H) significantly reduced the infarct volume compared to the untreated tMCAO group. Due to the majority of patients in clinical practice suffering from ischemic stroke that occupies white matter, we tested the therapeutic effect of RPR on white matter. We also evaluated the changes in infarct size in the white matter of tMCAO mice after the administration of RPR. The results showing the ischemic-induced injury in the tMCAO group elicited a prominent elevation in infarct volume in white matter when compared to the sham group. The administration of high-dose RPR (RPR-H) significantly reduced the infarct volume in white matter compared to the untreated tMCAO group (Supplementary Figure S2).

Histopathological examination revealed extensive cerebral tissue damage stemming from ischemia/reperfusion injury. The HE stain showed the presence of enlarged or shrunken neurons (black arrow), multiple vacuole-filled areas (green arrow) were seen in brain tissues, and the distribution of cells became scattered and disorganized. Nissl stain showed Nissl bodies diminished (red arrow) in neurons. Compared with the tMCAO mice, treatment with RPR was effective in alleviating the pathological abnormalities caused by ischemia/reperfusion (Figure 4C).

The neurological score acted as an indicator to quantify the degree of neurological deficit present in the mice. As shown in Figure 4D, tMCAO mice strongly altered the neural behavior of mice and showed the highest neurological score. Treatment with RPR-H significantly reduced the neurological score with better neural behavior compared with the tMCAO mice.

The survival rate at 72 h post-reperfusion results indicated that RPR could increase the survival rate of mice within 72 h after tMCAO, suggesting that RPR also had a significant long-term beneficial effect post-reperfusion (Supplementary Figure S3).

The disruption of the blood–brain barrier (BBB) is a main pathophysiological feature of ischemic stroke [16]. Tight junction (TJ) is one of the ways in which cells come into contact with each other; TJs are the key element of the BBB [17]. ZO-1 [18], Occludin [19,20], and Claudin-5 [21] are important for TJs in BBB; thus, we examined the expression of ZO-1, Occludin, and Claudin-5 by Western blot to investigate the effect of RPR on BBB integrity. The expression levels of ZO-1, Occludin, and Claudin-5 were significantly downregulated in tMCAO mice, indicating the obvious decrease in BBB integrity in tMCAO mice, while RPR treatment significantly upregulated the levels of these three proteins in tMCAO mice (Figure 4E,F). In addition, treatment with RPR significantly decreased the level of IL-6 and IL-1β in tMCAO mice (Figure 4G,H). These results indicated that RPR exhibited protective effects on cerebral ischemia–reperfusion injury.

### 3.4. RPR Exerts Protective Effects on Ischemic Stroke Through MAPK Signaling Pathway

**Figure 5:**
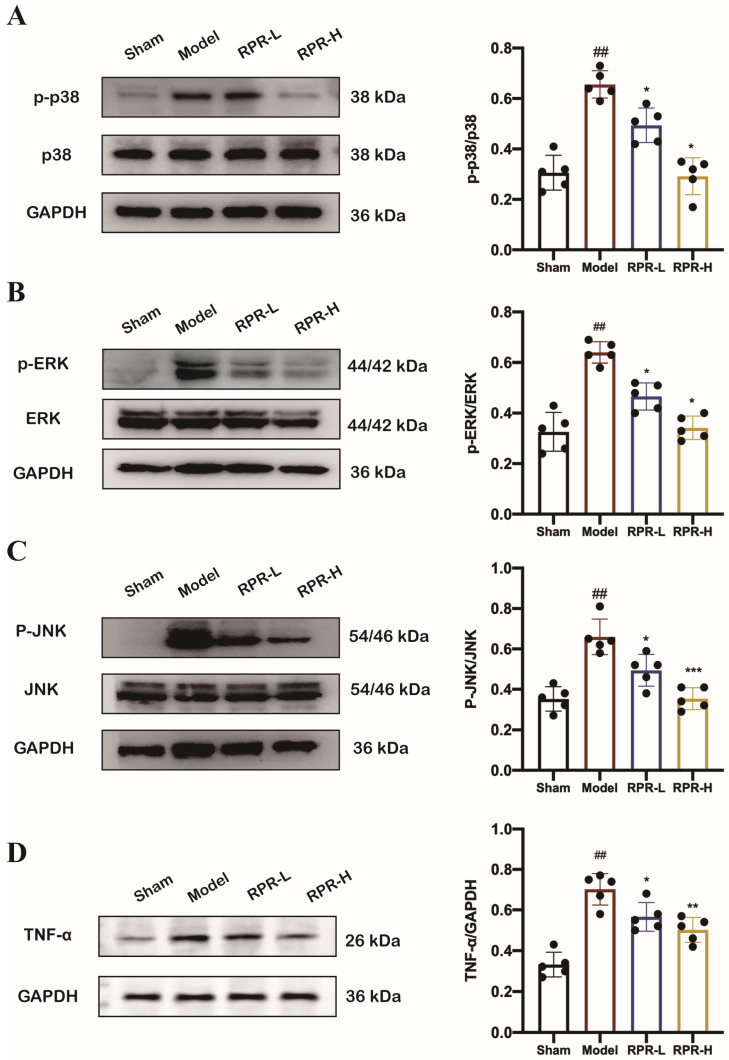
RPR inhibited stroke via the MAPK signaling pathway. Western blotting and quantification analysis of p-p38, p38 (**A**), p-ERK, ERK (**B**), p-JNK, JNK (**C**), and TNF-α (**D**) in the brain tissue of tMCAO mice. *n* = 5. The data are presented as mean ± SD. *## p* < 0.01 vs. Sham group, * p < 0.05, ** p < 0.01, *** p < 0.001 vs. Model group.

On the basis of the KEGG enrichment analysis of network pharmacology, the MAPK signaling pathway was one of the main signaling pathways of RPR in treating ischemic stroke, and the PPI network displayed the main target as MAPK3 (ERK 1) and MAPK14 (p38). Utilizing Western blot, we confirmed the validity of the proposed mechanism. As shown in Figure 5A–C, there was an obvious increase in the expression of phosphorylation of ERK, p38, and JNK in tMCAO mice. Meanwhile, these effects were largely attenuated by RPR treatment. Considering the pivotal role of TNF-α among key targets and its significance in ischemic stroke pathology, we subsequently delved into examining the changes in protein expression levels. As shown in Figure 5D, treatment with RPR led to a notable reduction in TNF-α protein levels in tMCAO mice. In addition, we validated the changes in MAPK3, MAPK14, and JNK targets in infarct size in the white matter of tMCAO mice after the administration of RPR. The Western blot experiment showed that RPR treatment significantly reduce the upregulation of phosphorylation of ERK, p38, and JNK in the white matter of tMCAO mice brain. (Supplementary Figure S4). These results demonstrated that RPR exerts a protective effect on ischemic stroke at least in part by down-regulating MAPK signaling pathways and TNF-α.

### 3.5. Molecular Docking

**Figure 6:**
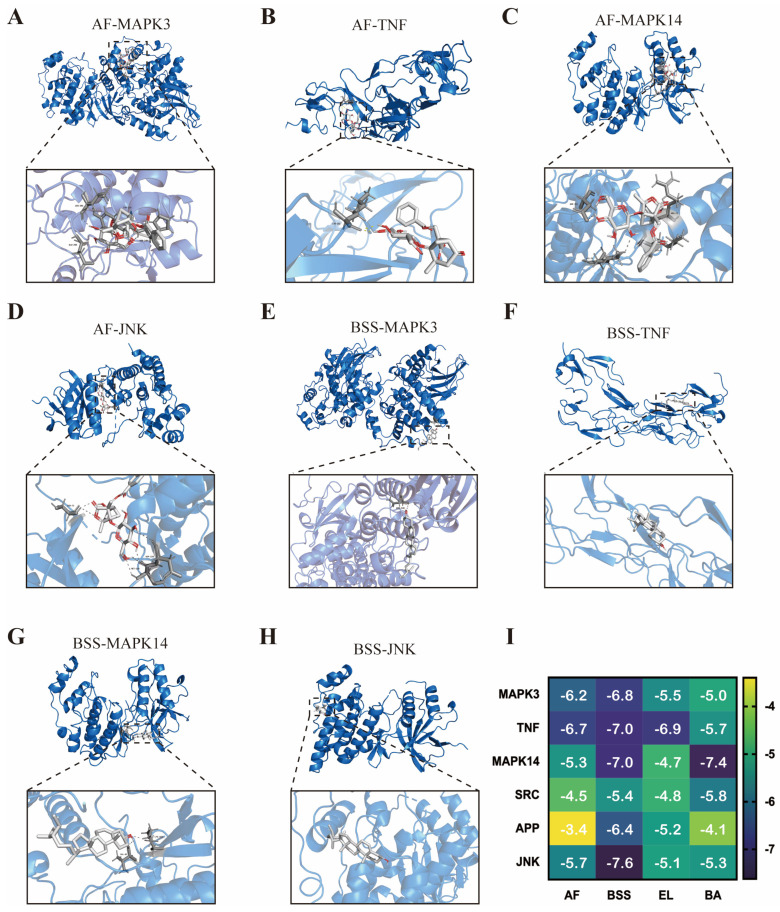
Molecular docking: (**A**–**D**) The results of molecular docking of MAPK3 (**A**), TNF (**B**), MAPK14 (**C**), and JNK (**D**) with AF. (**E**–**H**) The results of molecular docking of MAPK3 (**E**), TNF (**F**), MAPK14 (**G**), and JNK (**H**) with BSS. (**I**) Heat maps of the docking binding energy of the top 6 core targets with the top 4 active compounds in RPR.

In order to further investigate the main active ingredients in RPR, the five key components of RPR were selected for molecular docking validation with the four key targets. All docking combinations exhibited docking energies below −5.5 Kcal/mol, indicating successful and effective docking. Among the components and targets, the binding energy of AF and BSS with the targets was mostly higher than the other three ingredients. AF showed high binding affinity with MAPK3, TNF-α, MAPK14, and JNK (Figure 6A–D), and BSS exhibited strong with MAPK3, TNF-α, MAPK14, and JNK (Figure 6E–H). The heat map visualization was utilized to calculate and represent the docking energies for all combinations (Figure 6I). Through molecular docking results, we obtained two active ingredients (AF and BSS) in RPR that may exert anti-stroke effects; further in vitro experiments will evaluate these two components.

### 3.6. RPR and Its Active Ingredients Inhibit Platelet Activation

The pathophysiology of ischemic stroke includes both inflammatory and thrombogenic, and it is increasingly considered a thrombo-inflammatory disease [22,23]. Mounting experimental evidence suggests that the abnormal activation of platelets results in stroke [4]. Our preliminary research indicated that RPR significantly inhibits the formation of cerebral tissue thrombosis and reduces the activation of platelets in cerebral tissue thrombosis in tMCAO mice. Thus, we assessed the influence of RPR and its active ingredients on platelet activation in vitro.

**Figure 7:**
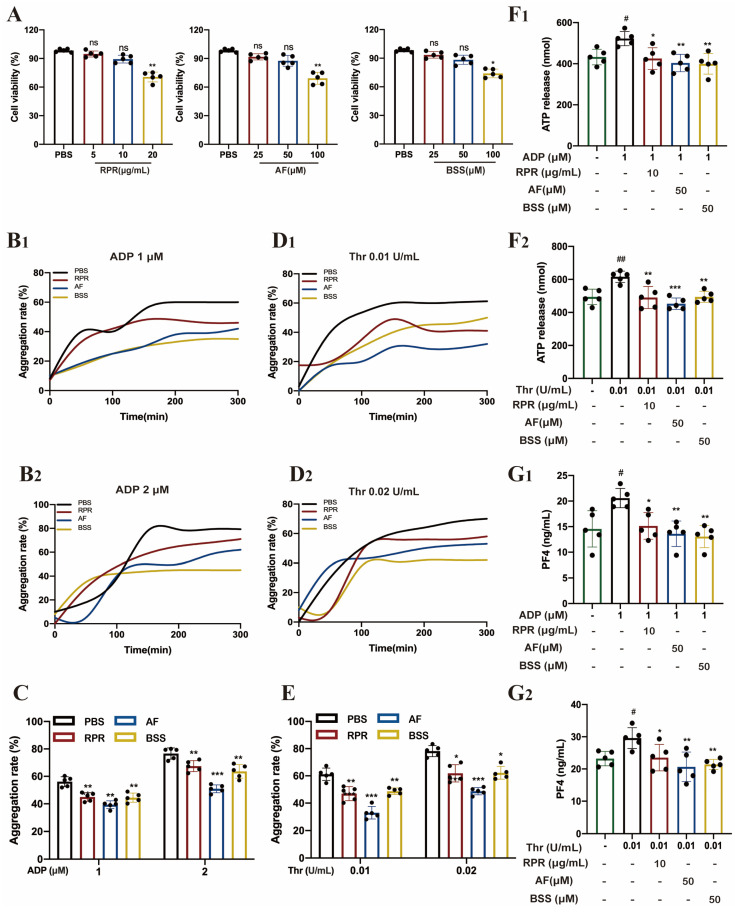
Active compounds of RPR inhibit agonist-induced platelet aggregation and granules release: (**A**) The effects of RPR and the two active compounds, including AF and BSS on cell viability of platelets. (**B**–**E**) The effects of RPR and the two active compounds, including AF and BSS on aggregation induced by thrombin (**B1**,**B2**,**C**) and ADP (**D1**,**D2**,**E**). (**F**) The effects of RPR and the two active compounds, including AF and BSS, on the release of ATP secretion induced by thrombin (**F1**) and ADP (**F2**). (**G**) The effects of RPR and the two active compounds, including AF and BSS on the release of PF4 induced by thrombin (**G1**) and ADP (**G2**); *n* = 5. The data are presented as mean ± SD. * p < 0.05, ** p < 0.01, *** p< 0.001, *# p* < 0.05, *## p* < 0.01, ns, no significance.

**Figure 8:**
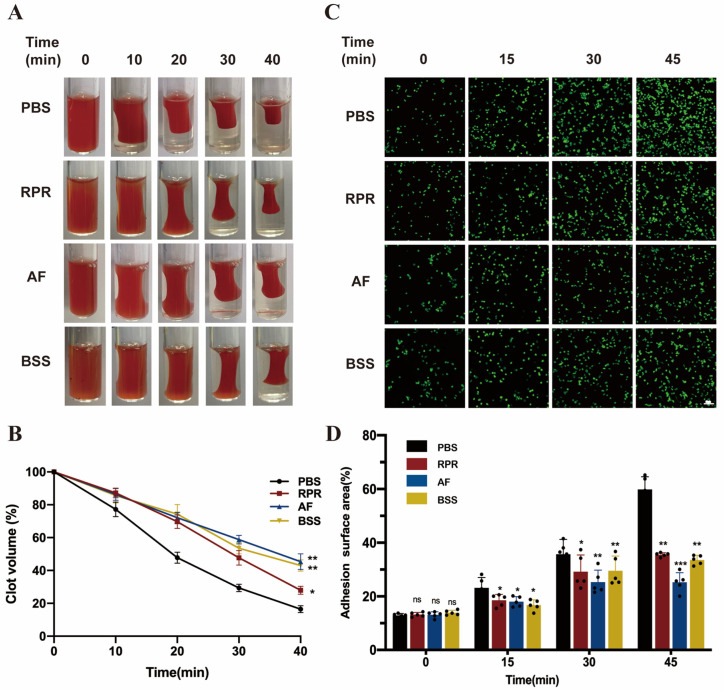
Active compounds of RPR inhibit platelet clot retraction and adhesion: (**A**) The effects of RPR and the two active compounds, including AF and BSS on platelet clot retraction within 40 min. (**B**) Statistics of the volume of clot retraction. (**C**) The effects of RPR and the two active compounds, including AF and BSS, on platelet adhesion within 45 min. (**D**) Statistics of the area of platelet adhesion, *n* = 5. Results were quantified and presented as mean ± SD, * p < 0.05, ** p < 0.01, *** p< 0.001 vs. PBS group, ns, no significance.

The two active compounds AF and BSS in PRP were confirmed through HPLC analysis (Supplementary Figure S2). Based on the CCK8 results, the doses of RPR, AF, and BSS reached 10 μg/mL, 50 μM, and 50 μM, respectively, and showed no significant cytotoxicity to platelets (Figure 7A). Therefore, these doses were used for further experiments. First of all, we found that RPR, AF, and BSS showed pronounced suppressive effects on the aggregation of platelets induced by ADP (Figure 7B1,B2,C) and thrombin (Figure 7D1,D2,E), which is one of the characteristic events of platelet activation. Furthermore, we tested the effect of RPR, AF, and BSS on platelet granule release, including α-granule release and dense granules, which is another core event involved in platelet activation [24,25,26]. As shown in Figure 7F,G, RPR, AF, and BSS significantly inhibited the release of platelet particles, including PF4 (the marker of α-granules) release and ATP (the marker of dense granules), stimulated by thrombin (Figure 7F and Supplementary Figure S3) and ADP (Figure 7G and Supplementary Figure S3). Given that the αIIbβ3 outside-in signaling drives processes essential for hemostasis, including platelet spreading and clot retraction, we examined the effect of RPR, AF, and BSS on platelet spreading and clot retraction [27,28]. Our results demonstrated that, treated with RPR, AF and BSS inhibited platelet clot retraction (Figure 8A,B); moreover, RPR, AF and BSS significantly decreased the area of adhesion of platelets on fibrinogen (Figure 8C,D) compared with the PBS group.

Due to previous experiments indicating the therapeutic effect of RPR on ischemic stroke in mice through the MAPK signaling pathway, we hypothesize that RPR and its active ingredients inhibit platelet activation through the MAPK signaling pathway to improve ischemic stroke. Western blot analysis was used to detect the MAPK signaling pathway in platelets treated with RPR and its active ingredients. The results showed that RPR and its active components significantly inhibited the activation of the MAPK signaling pathway after platelet activation, indicating that RPR and its active components likely inhibit platelet activation by affecting MAPK signal transduction (Supplementary Figure S7). 

In summary, the results suggested that RPR and it active ingredients (AF and BSS) exert inhibitory effects on multiple processes of agonist-induced platelet activation through the MAPK signaling pathway.

## 4. Discussion

Ischemic stroke emerges as the most prevalent form of stroke, characterized by a sudden decrease or complete interruption of blood flow to the brain, often caused by thrombosis or embolism within the cerebral blood vessels [29]. Currently, the main approaches to treating ischemic stroke are two categories: the utilization of the intravenous tissue plasminogen activator (tPA) and mechanical thrombectomy as therapeutic modalities [30]. RPR, as a TCM, effectively promotes blood circulation and eliminates blood stasis [31]. RPR has demonstrated efficacy in treating a range of cardiovascular and cerebrovascular ailments, particularly atherosclerosis [32], coronary heart disease [33], and myocardial infarction, but its therapeutic effect on stroke has not been fully explored. Our research aimed to elucidate the protective mechanism of RPR against ischemic stroke, leveraging a multidisciplinary approach encompassing network pharmacology, molecular docking simulations, and experimental confirmation.

Because the direct cause of ischemic stroke is the obstruction of the MCA, we established the tMCAO animal model to explore the effect of RPR on ischemic stroke. Neurological impairment and cerebral infarction resulting from decreased cerebral blood flow were the clinical symptoms of brain injury [34]. The in vivo experiments demonstrated a pronounced decrease in cerebral infarction and neurological deficits in tMCAO mice that were pretreated with a high dose of RPR for three consecutive days. Pathology analysis showed that RPR significantly inhibited the aforementioned histological damage in tMCAO mice, including the pyknosis of neuronal cell nucleus, the loss and edema of brain tissue, the atrophy of nerve cells, and the reduction in Nissl bodies in neurons. BBB dysfunction is a salient pathological hallmark in ischemic stroke, characterized by increased permeability caused by the disintegration of tight junction protein complexes, cerebral edema caused by dysfunction in ion transporter systems, coupled with subsequent inflammatory injury instigated by infiltrating leukocytes, and is a notable pathological characteristic of ischemic stroke [35]. Western blot results indicated that RPR relieved the dysfunction of BBB in tMCAO mice.

Recent studies have demonstrated that the powerful capabilities of network pharmacology in identifying potential active compounds and protein targets have provided novel perspectives and solutions for clarifying the pharmacological mechanisms of Traditional Chinese Medicine (TCM) [36]. Network pharmacology research revealed that RPR contains 13 active ingredients and 1434 targets, among which 128 were shared between RPR and ischemic stroke. Lipid and atherosclerosis, the MAPK signaling pathway, the Rap1 signaling pathway, and HIF-1 signaling pathways were enriched in the KEGG analysis of these targets as a top ranked signaling pathway. Furthermore, among the 128 overlapping targets, five core targets have been identified and validated through molecular docking, namely MAPK3 (ERK 1), TNF-α, MAPK14 (p38), SRC, and JNK. Notably, MAPK3, as a part of the MAPK signaling pathway, emerged as a pivotal target within the protein–protein interaction network, characterized by its prominent degree. The MAPK signaling pathway, which has garnered extensive research attention, plays a pivotal role in modulating inflammatory reactions, cytokine production, and apoptotic processes following a stroke event [37,38]. TNF-α serves as a pivotal regulator in diverse biological processes, encompassing inflammation, autoimmune responses, allergic manifestations, and antitumor mechanisms [39]. In ischemic stroke, local inflammation and thrombosis are accompanied by elevated levels of TNF-α [40,41,42]. In this study, we preliminarily observed that RPR significantly downregulate the MAPK signaling pathways and TNF-α. However, the current research results are only preliminary, so it is necessary to use inhibitors of the MAPK signaling pathway and TNF-α, as well as knockout mouse models of the MAPK signaling pathway and TNF—α genes, in order to further verify the role of RPR in the MAPK signaling pathway and key targets such as TNF—α in stroke mice. Molecular docking was performed between the bioactive ingredients screened through network pharmacology and essential proteins, revealing that albiflorin (AF) and β-sitosterol (BSS) exhibited the highest docking scores with MAPK3, TNF-α, MAPK14, and JNK. This indicated that the active ingredients of RPR primarily bind to these targets to ameliorate ischemic stroke.

The activation of platelets is pivotal in the development of thrombotic disorders, where abnormal platelet activation cause thrombosis and vessel occlusion, ultimately resulting in stroke. Therefore, antiplatelet therapy is a crucial strategy in stroke management [43,44,45,46]. We evaluated the effect of RPR and two active ingredients (AF and BSS) on platelet activation. The in vitro experiment results demonstrated RPR and two active ingredients (AF and BSS) not only inhibited platelet aggregation induced by ADP and thrombin but also exhibited obvious inhibitory effects on the release of platelet α-particles and dense particles, which are the important markers of platelet activation. Furthermore, integrin αIIbβ3 serves as the surface receptor of platelets, and its activation is considered as the ultimate common pathway for platelet activation, participating in the bidirectional signaling during platelet activation. This includes the inside–out signal represented by platelet adhesion events and the outside–in signal represented by platelet clot retraction. Our results revealed that RPR and its two active ingredients (AF and BSS) reduced the area of platelet spreading and inhibited platelet clot retraction. The discovery of RPR’s ability to inhibit platelet activation and thrombosis makes it a potential drug for the prevention and treatment of thrombotic diseases such as atherosclerosis, coronary heart disease, stroke, and other related conditions. Meanwhile, RPR could be applied in various ways to treat human cardiovascular diseases. For instance, it can serve as an ingredient in cardiovascular medications and be combined with other antiplatelet drugs (such as Aspirin and Clopidogrel) to enhance their antiplatelet effects. Additionally, RPR can be used in the preparation of health foods or functional foods for the prevention of cardiovascular diseases.

The limitations of this study are as follows: (1) The limitations of animal experimental design: Firstly, our experiment did not use randomization and blinding methods, which may lead to bias in the selection of experimental animals due to subjective judgments of researchers and individual differences, thereby reducing the reliability and accuracy of the experiment. Secondly, individual differences between mice can affect the progression of infarction and the results of ischemic stroke experiments. Thirdly, the sample size of animals is relatively small. It may lead to a decrease in statistical ability, making the results more susceptible to random errors or sample bias, resulting in higher standard deviations for some experimental results such as RPR improving neurological scores and reducing infarct volume. Lastly, the tMCAO model has a faster perfusion rate than that of human Central Africa and cannot integrate other complications of human stroke (such as hypertension, diabetes, hyperlipidemia), so it has limitations in completely replicating human stroke pathology. However, our current research results preliminarily demonstrate that RPR does have a significant improvement effect on ischemic stroke. In future studies, we will follow the principles of randomization and blinding, increase sample size and use advanced imaging techniques to improve the reliability and accuracy of the experiment. (2) The limitations of network pharmacology: The computational methods for predicting drug therapy targets using network pharmacology have certain limitations. First of all, the database information lacks completeness and accuracy. The research of network pharmacology highly relies on specific databases, and the information in these databases mainly comes from the published literature. Due to limitations in research hotspots and disease types, there may be biases in the inclusion of database information, which can affect the completeness and accuracy of the results. Secondly, there is the lack of experimental validation. The calculation methods of network pharmacology are mainly based on theoretical prediction and data analysis, but, due to the gap between theoretical prediction and experimental verification, the predicted results may not be consistent with the experimental results. To overcome these limitations, it is necessary to further optimize screening criteria, data processing methods, and strengthen experimental validation, such as repeating experiments or increasing sample sizes to improve the reliability and accuracy of experimental results. (3) The limitations of molecular docking: Molecular docking has some limitations in predicting true binding affinity and biological activity [47]. The molecular docking calculation model cannot fully replicate the real environment inside the organism [48]. And due to the influence of factors such as intermolecular interactions, spatial conformational changes, and energy matching on binding affinity, molecular docking simulations have limitations in accurately predicting binding affinity [49,50]. To overcome these limitations, future experiments should further adapt to SPR and MST experiments, in order to more comprehensively and accurately demonstrate the therapeutic effect of RPR on ischemic stroke through the MAPK signaling pathway, providing more valuable information for the treatment, drug development, and clinical application of ischemic stroke.

In summary, we systematically assessed the pharmacodynamic effects of RPR against ischemic stroke, utilizing an integrated strategy integrating network pharmacology and experimental confirmation. Our results suggest that RPR treats ischemic stroke by diminishing the cerebral infarct volume, enhancing neurological function recovery and restoring the BBB via the MAPK signaling pathway. Additionally, RPR and its two active ingredients (AF and BSS) exhibited the inhibitory effects on platelet activation, indicating that RPR alleviates ischemic stroke, at least partially, achieved by affecting platelet function. Our findings not only highlighted the potential clinical value of RPR in the field of anti-platelet therapy and provided broad prospects for its application in the treatment of cardiovascular diseases but also provided ideas for new drug candidates for ischemic stroke.

## 5. Conclusions

**Figure 9:**
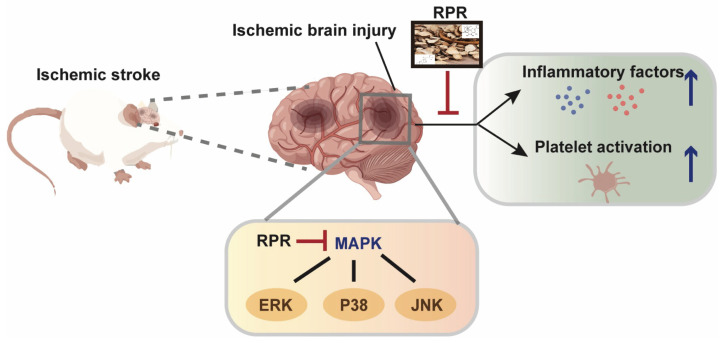
The proposed mechanism of RPR therapeutic effects on ischemic stroke. RPR alleviates ischemic stroke by down-regulating MAPK pathways; furthermore, the protective effect may be partly due to the anti-platelet function of its active compounds.

Drawing upon a comprehensive approach encompassing network pharmacology analysis, molecular docking simulations, and experimental validation, we have demonstrated that RPR showed significant therapeutic effects on ischemic stroke via the MAPK signaling pathway. Furthermore, RPR’s inhibitory effect on platelet aggregation, particle release, spreading and clot retraction indicated that RPR attenuates cerebral ischemia partly due to its anti-platelet function (Figure 9). Our work provides new strategies and drug candidates for ischemic stroke and anti-platelet therapy.

## Figures and Tables

**Table 1:** Information of top 10 core targets.

No.	Target	Degree	Betweenness Centrality	Closeness Centrality
1	MAPK3	62	2695	0.0056
2	TNF	58	2107	0.0049
3	MAPK14	50	1897	0.0047
4	SRC	36	1243	0.0045
5	JNK	32	1211	0.0044
6	Nox4	25	849	0.0043
7	Hif1a	20	787	0.0043
8	Xdh	15	750	0.0042
9	Snca	8	671	0.0040
10	Ppara	6	594	0.0041

**Table 2:** Clusters of common targets.

Cluster	Gene Count	Number of Nodes	Number of Edges
1	48	48	144
2	46	46	84
3	34	34	41

## Data Availability

Data will be provided upon request in Supplementary Materials.

## Supplementary Materials

The following supporting information can be downloaded at: https://www.mdpi.com/article/10.3390/nu16244409/s1, Figure S1: PPI analysis of the RPR in the treatment of stroke targets; Figure S2: The quantification results of brain infarction volume of white matter (*n* = 5); Figure S3: Survival rate of mice after 72 h of tMCAO reperfusion (*n* = 9); Figure S4: RPR inhibited stroke via the MAPK signaling pathway; Figure S5: HPLC identification of RPR, AF and BSS. HPLC identification of RPR, AF and BSS. BSS (B) at 2.921 min, AF (A) at 11.578 min; Figure S6: Active compounds of RPR inhibit agonist-induced platelet aggregation and granules release; Figure S7: RPR, AF and BSS inhibit platelet activation through the MAPK signaling pathway; Table S1: Main active compounds in RPR; Table S2: Ischemic stroke targets of RPR from GeneCards databases; Table S3: Ischemic stroke targets of RPR from OMIM databases; Table S4: Ischemic stroke targets of RPR from DisGeNET databases; Table S5: Total ischemic stroke targets of RPR.
