# Supplementary material for: Investigation into the Potential Mechanism of Radix Paeoniae Rubra Against Ischemic Stroke Based on Network Pharmacology
Source: Nutrients. 2024 Dec 23;16(24):4409. doi: 10.3390/nu16244409 (PMC11678013; doi:10.3390/nu16244409)
Supplement: Supplementary file 1 [file nutrients-16-04409-s001.zip › Supplementary file/nutrients-3246443-supplementary.pdf]

Supplementary Materials:

Figure S1. PPI analysis of the RPR in the treatment of stroke targets. (A) PPI network constructed by STITC database; (B) The core targets of the 128 common targets ranked by degree value.

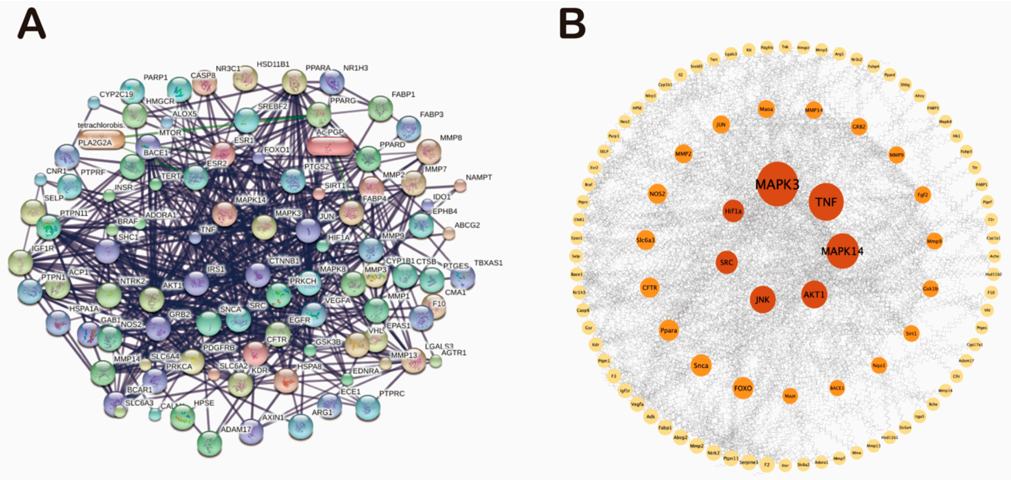

Figure S2. The quantification results of brain infarction volume of white matter (n=5).

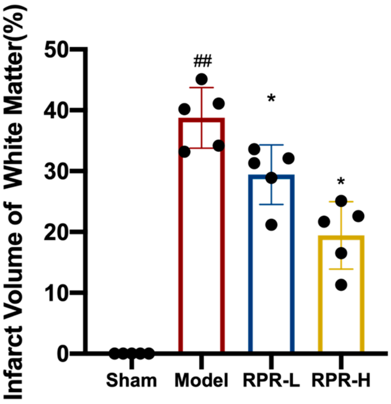

Figure S3. Survival rate of mice after 72 hours of tMCAO reperfusion.(n= 9)

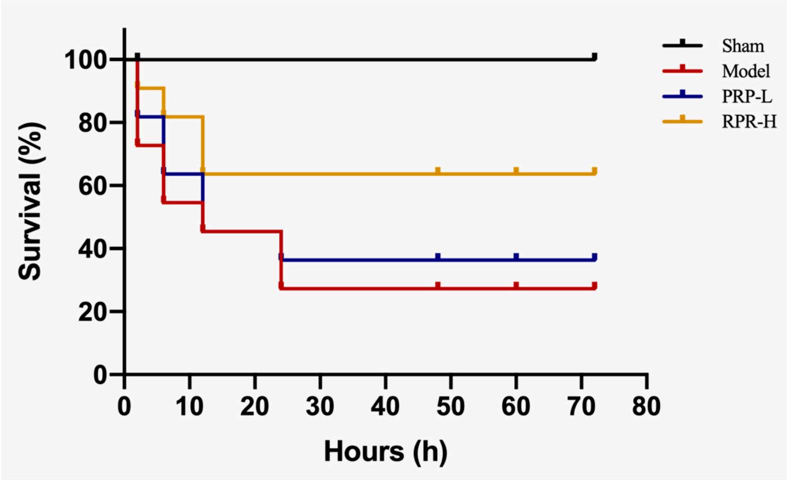

Figure S4. RPR inhibited stroke *via* the MAPK signaling pathway. Western blotting and quantification analysis of p-p38, p38 (A), p-ERK, ERK (B), p-JNK, JNK (C), and TNF-α (D) in white matter of tMCAO mice. n=5. The datas are presented as mean ± SD. ##$p < 0.01$, *vs.* Sham group, *$p < 0.05 < 0.01$ *vs.* Model group.

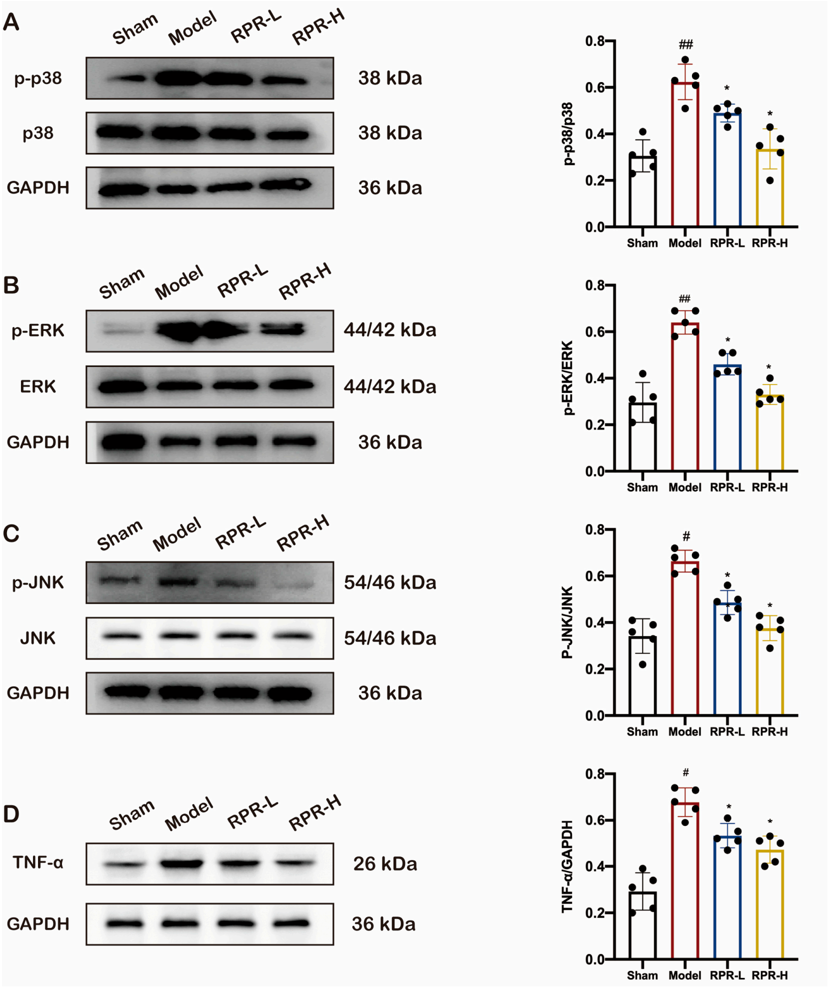

Figure S5. HPLC identification of RPR, AF and BSS. HPLC identification of RPR, AF and BSS. BSS (B) at 2.921 min, AF (A) at 11.578 min.

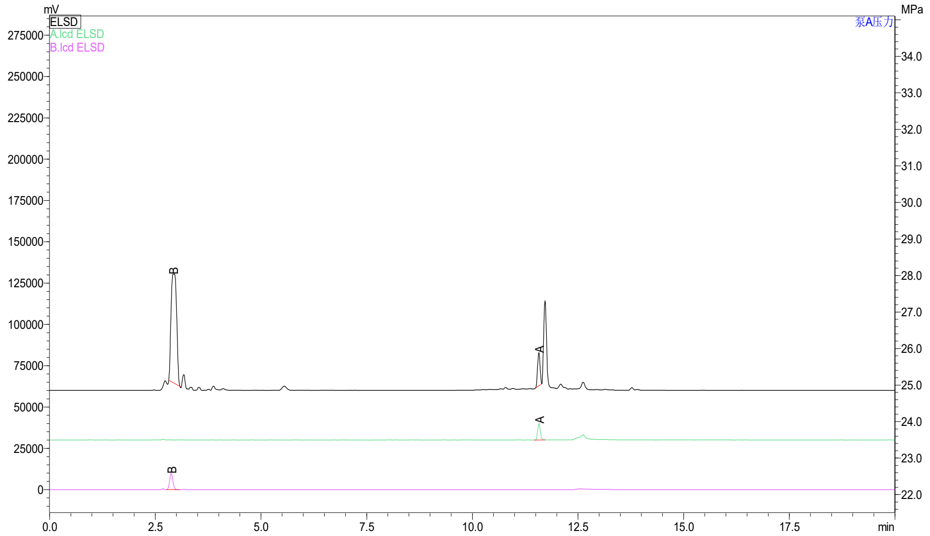

Figure S6. Active compounds of RPR inhibit agonist-induced platelet aggregation and granules release (A-B) The effects of RPR and the two active compounds, including AF and BSS on the release of ATP secretion induced by ADP (A) and thrombin (B). (C-D) The effects of RPR and the two active compounds, including AF and BSS on the release of PF4 induced by ADP (A) and thrombin (B); n=5. The data are presented as mean ± SD. **p* < 0.05, ***p* < 0.01, #*p* < 0.05, ##*p* < 0.01.

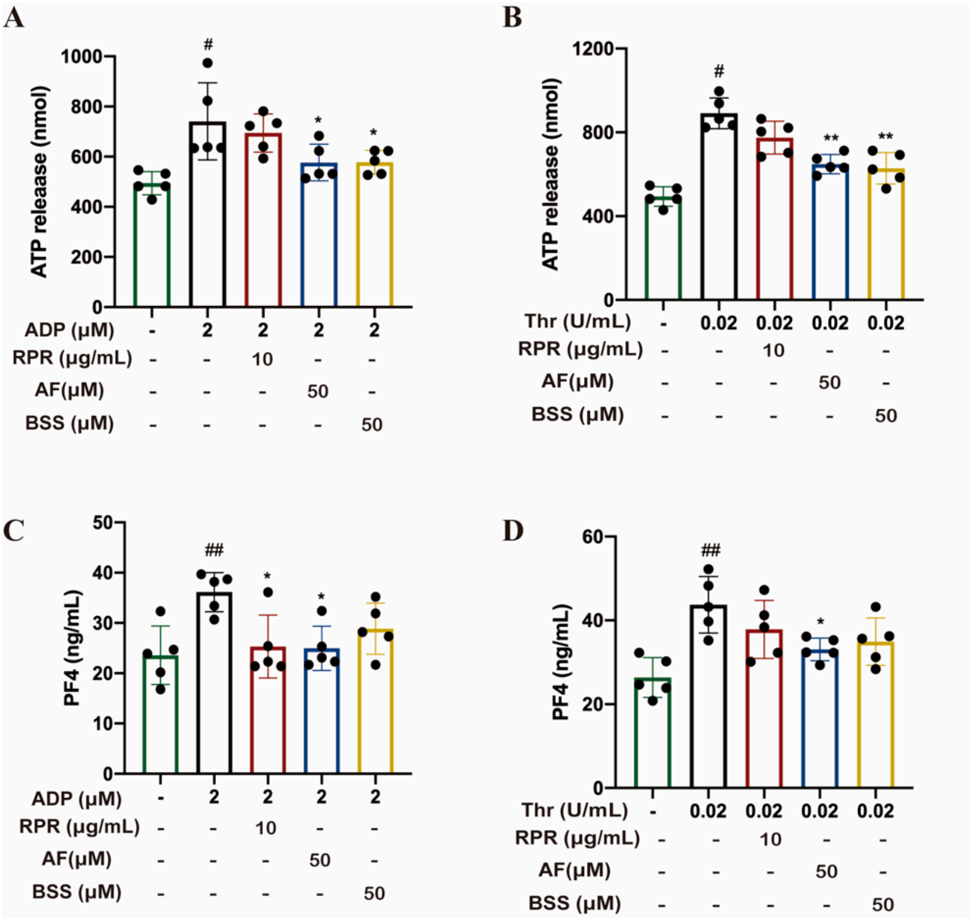

Figure S7. RPR , AF and BSS inhibit platelet activation through the MAPK signaling pathway. Western blotting and quantification analysis of p-p38, p38

(A), p-ERK, ERK (B)and p-JNK, JNK (C) of platelet. n=3. The data are presented as mean ± SD. ##$p < 0.01$, *vs.* Sham group, *$p < 0.05$, **$p < 0.01$ *vs.* Model group.

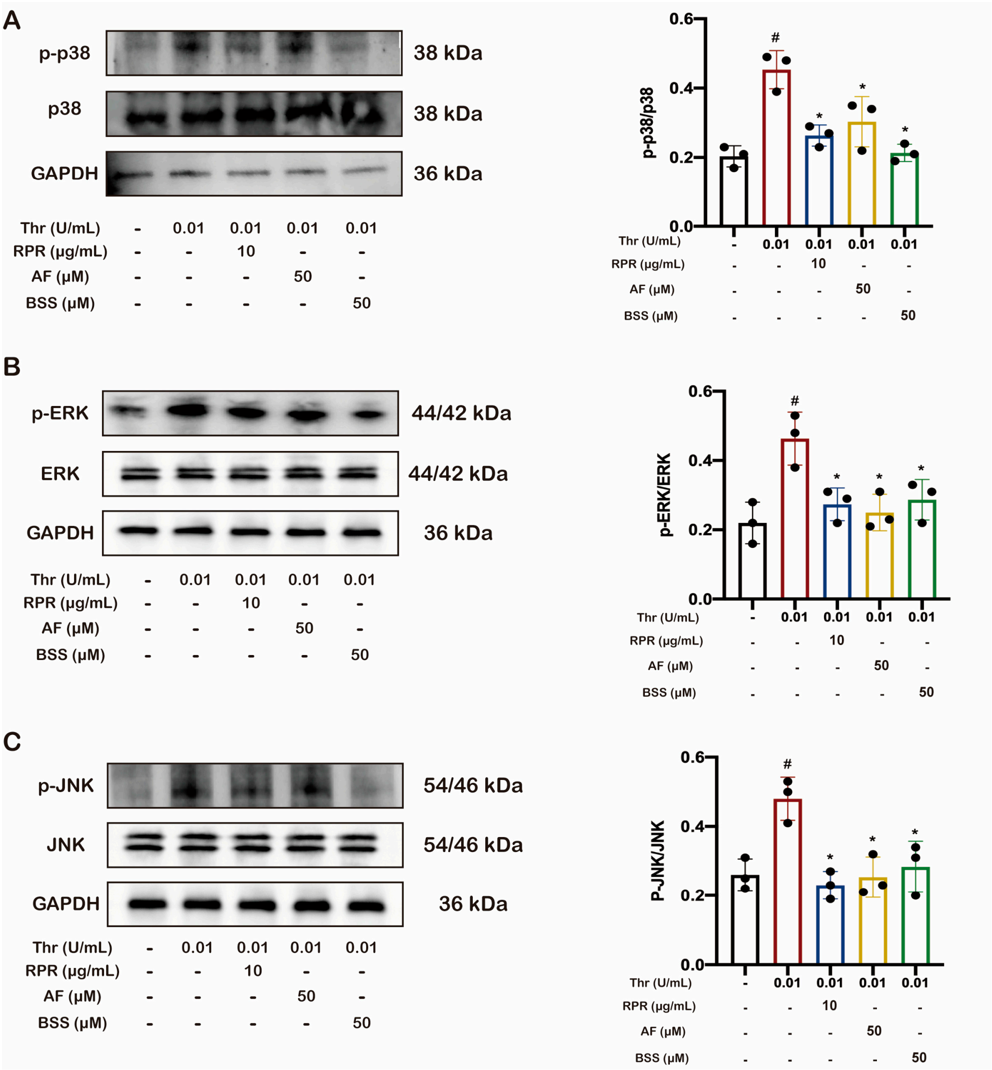

Table S1. Main active compounds in RPR.

No.	MOL Number	Molecule Name	Molecule Weight	Molecular Structure
1	MOL001002	ellagic acid	302.20	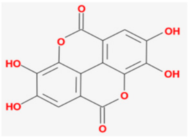
2	MOL001918	paeoniflorgenone	318.35	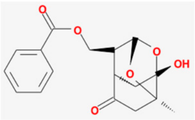
3	MOL001921	Lactiflorin	462.49	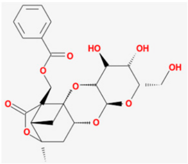

**4**	MOL001924	paeoniflorin	480.51	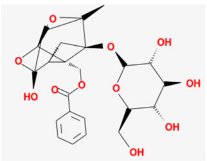
**5**	MOL002714	baicalein	270.25	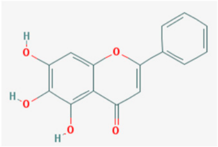
**6**	MOL002776	Baicalin	446.39	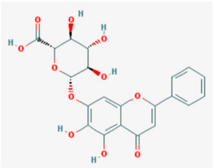
**7**	MOL000358	beta-sitosterol	414.79	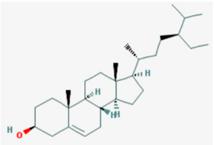
**8**	MOL000359	sitosterol	414.79	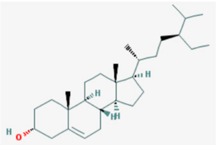
**9**	MOL004355	Spinasterol	412.77	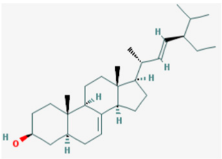
**10**	MOL000449	Stigmasterol	412.77	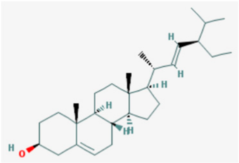
**11**	MOL006999	stigmast-7-en-3-ol	414.79	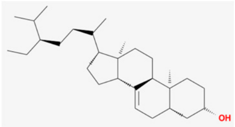
**12**	MOL007004	Albiflorin	480.51	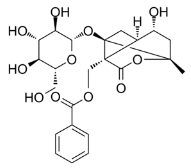

**13**	MOL002883	Ethyl oleate (NF)	310.58	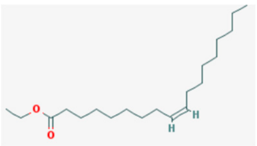

Table S2. Ischemic stroke targets of RPR from GeneCards databases.

Gene	Name
NOTCH3	Notch Receptor 3
F5	Coagulation Factor V
ACE	Angiotensin I Converting Enzyme
F2	Coagulation Factor II, Thrombin
NOS3	Nitric Oxide Synthase 3
MTHFR	Methylenetetrahydrofolate Reductase
MT-TL1	Mitochondrially Encoded TRNA-Leu (UUA/G) 1
ALOX5AP	Arachidonate 5-Lipoxygenase Activating Protein
GP1BA	Glycoprotein Ib Platelet Subunit Alpha
PRKCH	Protein Kinase C Eta
RNF213	Ring Finger Protein 213
FBN1	Fibrillin 1
COL4A1	Collagen Type IV Alpha 1 Chain
BDNF-AS	BDNF Antisense RNA
CRP	C-Reactive Protein
FGB	Fibrinogen Beta Chain
APOE	Apolipoprotein E
IL6	Interleukin 6
ADA2	Adenosine Deaminase 2
APOB	Apolipoprotein B
MALAT1	Metastasis Associated Lung Adenocarcinoma Transcript 1
MIAT	Myocardial Infarction Associated Transcript
TNF	Tumor Necrosis Factor
MT-CYB	Mitochondrially Encoded Cytochrome B
MT-ND1	Mitochondrially Encoded NADH:Ubiquinone Oxidoreductase Core Subunit 1
LDLR	Low Density Lipoprotein Receptor
LOC132090228	Neanderthal Introgressed Variant-Containing Enhancer Experimental_34691
MT-TK	Mitochondrially Encoded TRNA-Lys (AAA/G)
SERPINE1	Serpin Family E Member 1
PLAT	Plasminogen Activator, Tissue Type
MT-TS1	Mitochondrially Encoded TRNA-Ser (UCN) 1
CDKN2B-AS1	CDKN2B Antisense RNA 1
MT-ND5	Mitochondrially Encoded NADH:Ubiquinone Oxidoreductase Core Subunit 5
MEG3	Maternally Expressed 3
HTRA1	HtrA Serine Peptidase 1
APP	Amyloid Beta Precursor Protein
ACSL4	Acyl-CoA Synthetase Long Chain Family Member 4
PDE4D	Phosphodiesterase 4D
MT-CO3	Mitochondrially Encoded Cytochrome C Oxidase III
PIK3CA	Phosphatidylinositol-4,5-Bisphosphate 3-Kinase Catalytic Subunit Alpha
CBS	Cystathionine Beta-Synthase
COL4A2	Collagen Type IV Alpha 2 Chain
POLG	DNA Polymerase Gamma, Catalytic Subunit
ACTA2	Actin Alpha 2, Smooth Muscle
MT-TI	Mitochondrially Encoded TRNA-Ile (AUU/C)
CD36	CD36 Molecule (CD36 Blood Group)
GLA	Galactosidase Alpha
BDNF	Brain Derived Neurotrophic Factor
MT-TL2	Mitochondrially Encoded TRNA-Leu (CUN) 2
MMP9	Matrix Metallopeptidase 9
IL1B	Interleukin 1 Beta
MT-CO1	Mitochondrially Encoded Cytochrome C Oxidase I

POLGARF	POLG Alternative Reading Frame
VWF	Von Willebrand Factor
MT-TP	Mitochondrially Encoded TRNA-Pro (CCN)
NPPA	Natriuretic Peptide A
SERPINC1	Serpin Family C Member 1
AGT	Angiotensinogen
MT-ND6	Mitochondrially Encoded NADH:Ubiquinone Oxidoreductase Core Subunit 6
SELP	Selectin P
NPPB	Natriuretic Peptide B
H19	H19 Imprinted Maternally Expressed Transcript
MT-ND4	Mitochondrially Encoded NADH:Ubiquinone Oxidoreductase Core Subunit 4
FGA	Fibrinogen Alpha Chain
IL10	Interleukin 10
TREX1	Three Prime Repair Exonuclease 1
MT-ATP6	Mitochondrially Encoded ATP Synthase Membrane Subunit 6
PON1	Paraoxonase 1
MT-CO2	Mitochondrially Encoded Cytochrome C Oxidase II
JAK2	Janus Kinase 2
APOH	Apolipoprotein H
LPA	Lipoprotein(A)
SELE	Selectin E
INS	Insulin
EDN1	Endothelin 1
F3	Coagulation Factor III, Tissue Factor
AGTR1	Angiotensin II Receptor Type 1
TLR4	Toll Like Receptor 4
ENG	Endoglin
ITGB3	Integrin Subunit Beta 3
ATRIP	ATR Interacting Protein
ATRIP-TREX1	ATRIP-TREX1 Readthrough
ALB	Albumin
CYP2C19	Cytochrome P450 Family 2 Subfamily C Member 19
HIF1A	Hypoxia Inducible Factor 1 Subunit Alpha
TP53	Tumor Protein P53
MTR	5-Methyltetrahydrofolate-Homocysteine Methyltransferase
PLA2G7	Phospholipase A2 Group VII
PPARG	Peroxisome Proliferator Activated Receptor Gamma
ICAM1	Intercellular Adhesion Molecule 1
HOTAIR	HOX Transcript Antisense RNA
THBD	Thrombomodulin
CST3	Cystatin C
LPL	Lipoprotein Lipase
ENO2	Enolase 2
GAS5	Growth Arrest Specific 5
ADAMTS13	ADAM Metallopeptidase With Thrombospondin Type 1 Motif 13
RPL36A-HNRNPH2	RPL36A-HNRNPH2 Readthrough
GUCY1A1	Guanylate Cyclase 1 Soluble Subunit Alpha 1
SCN5A	Sodium Voltage-Gated Channel Alpha Subunit 5
MAPT	Microtubule Associated Protein Tau
PTGS2	Prostaglandin-Endoperoxide Synthase 2
REN	Renin
EPO	Erythropoietin
TGFB1	Transforming Growth Factor Beta 1
MIR126	MicroRNA 126
KAT6B	Lysine Acetyltransferase 6B
VEGFA	Vascular Endothelial Growth Factor A
CCL2	C-C Motif Chemokine Ligand 2
GFAP	Glial Fibrillary Acidic Protein
ADIPOQ	Adiponectin, C1Q And Collagen Domain Containing
F7	Coagulation Factor VII
VCAM1	Vascular Cell Adhesion Molecule 1
LMNA	Lamin A/C

S100B	S100 Calcium Binding Protein B
MIR21	MicroRNA 21
APOA1	Apolipoprotein A1
ITGA2	Integrin Subunit Alpha 2
P2RY12	Purinergic Receptor P2Y12
MIR155	MicroRNA 155
IL1A	Interleukin 1 Alpha
DIAPH1	Diaphanous Related Formin 1
F13A1	Coagulation Factor XIII A Chain
ABCC6	ATP Binding Cassette Subfamily C Member 6
F12	Coagulation Factor XII
GRIN2B	Glutamate Ionotropic Receptor NMDA Type Subunit 2B
HBB	Hemoglobin Subunit Beta
MYH11	Myosin Heavy Chain 11
NLRP3	NLR Family Pyrin Domain Containing 3
IL18	Interleukin 18
TNNI3	Troponin I3, Cardiac Type
LOX	Lysyl Oxidase
SLC2A1	Solute Carrier Family 2 Member 1
SOD2-OT1	SOD2 Overlapping Transcript 1
MIR125A	MicroRNA 125a
HMGB1	High Mobility Group Box 1
MIR140	MicroRNA 140
PECAM1	Platelet And Endothelial Cell Adhesion Molecule 1
TNNT2	Troponin T2, Cardiac Type
PF4	Platelet Factor 4
RNF213-AS1	RNF213 Antisense RNA 1
MIR223	MicroRNA 223
MPO	Myeloperoxidase
VKORC1	Vitamin K Epoxide Reductase Complex Subunit 1
IL1RN	Interleukin 1 Receptor Antagonist
CERNA3	Competing Endogenous LncRNA 3 For MiR-645
MIR144	MicroRNA 144
MIR146A	MicroRNA 146a
ELN	Elastin
TTR	Transthyretin
PSEN1	Presenilin 1
CASP3	Caspase 3
SOD1	Superoxide Dismutase 1
CXCL12	C-X-C Motif Chemokine Ligand 12
KCNJ5	Potassium Inwardly Rectifying Channel Subfamily J Member 5
MYLK	Myosin Light Chain Kinase
PITX2	Paired Like Homeodomain 2
CXCL8	C-X-C Motif Chemokine Ligand 8
PLG	Plasminogen
HMOX1	Heme Oxygenase 1
ATP1A2	ATPase Na+/K+ Transporting Subunit Alpha 2
FGF2	Fibroblast Growth Factor 2
PRNP	Prion Protein (Kanno Blood Group)
EDNRA	Endothelin Receptor Type A
CREB1	CAMP Responsive Element Binding Protein 1
IL4	Interleukin 4
MAP2	Microtubule Associated Protein 2
KCNQ1	Potassium Voltage-Gated Channel Subfamily Q Member 1
NGF	Nerve Growth Factor
IGF1	Insulin Like Growth Factor 1
HSPA4	Heat Shock Protein Family A (Hsp70) Member 4
ESR1	Estrogen Receptor 1
GBA1	Glucosylceramidase Beta 1
PROZ	Protein Z, Vitamin K Dependent Plasma Glycoprotein
CETP	Cholesteryl Ester Transfer Protein
ACE2	Angiotensin Converting Enzyme 2

PON2	Paraoxonase 2
PIK3C2A	Phosphatidylinositol-4-Phosphate 3-Kinase Catalytic Subunit Type 2 Alpha
CKB	Creatine Kinase B
LTA	Lymphotoxin Alpha
BCL2	BCL2 Apoptosis Regulator
KNG1	Kininogen 1
SCN1A	Sodium Voltage-Gated Channel Alpha Subunit 1
THPO	Thrombopoietin
CYCS	Cytochrome C, Somatic
GRIN2A	Glutamate Ionotropic Receptor NMDA Type Subunit 2A
MMP3	Matrix Metallopeptidase 3
NR4A2	Nuclear Receptor Subfamily 4 Group A Member 2
GPT	Glutamic--Pyruvic Transaminase
RETN	Resistin
MECP2	Methyl-CpG Binding Protein 2
TTN	Titin
MIR17	MicroRNA 17
TUG1	Taurine Up-Regulated 1
SH2B3	SH2B Adaptor Protein 3
HMGCR	3-Hydroxy-3-Methylglutaryl-CoA Reductase
HDAC9	Histone Deacetylase 9
CYP2C9	Cytochrome P450 Family 2 Subfamily C Member 9
KRIT1	KRIT1 Ankyrin Repeat Containing
ENPP1	Ectonucleotide Pyrophosphatase/Phosphodiesterase 1
AKT1	AKT Serine/Threonine Kinase 1
F10	Coagulation Factor X
MIR143	MicroRNA 143
NES	Nestin
CALR	Calreticulin
CD14	CD14 Molecule
FLNA	Filamin A
DARS2	Aspartyl-TRNA Synthetase 2, Mitochondrial
CTSA	Cathepsin A
SOD2	Superoxide Dismutase 2
NOS1	Nitric Oxide Synthase 1
SULT1A3	Sulfotransferase Family 1A Member 3
ABCA1	ATP Binding Cassette Subfamily A Member 1
MIR150	MicroRNA 150
ADRB2	Adrenoceptor Beta 2
MIR145	MicroRNA 145
ANGPT1	Angiopoietin 1
MIR29A	MicroRNA 29a
GPX3	Glutathione Peroxidase 3
ACVRL1	Activin A Receptor Like Type 1
OLR1	Oxidized Low Density Lipoprotein Receptor 1
APOC3	Apolipoprotein C3
GDNF	Glial Cell Derived Neurotrophic Factor
TNFRSF11B	TNF Receptor Superfamily Member 11b
ALOX5	Arachidonate 5-Lipoxygenase
MIR146B	MicroRNA 146b
ADD1	Adducin 1
TMX2-CTNND1	TMX2-CTNND1 Readthrough (NMD Candidate)
PPBP	Pro-Platelet Basic Protein
SLC2A10	Solute Carrier Family 2 Member 10
ADM	Adrenomedullin
MIR142	MicroRNA 142
NKX2-5	NK2 Homeobox 5
NFE2L2	NFE2 Like BZIP Transcription Factor 2
SMAD3	SMAD Family Member 3
SPP1	Secreted Phosphoprotein 1
STAT3	Signal Transducer And Activator Of Transcription 3
MPL	MPL Proto-Oncogene, Thrombopoietin Receptor

ZFHX3	Zinc Finger Homeobox 3
ITGA2B	Integrin Subunit Alpha 2b
SIRT1	Sirtuin 1
MIR98	MicroRNA 98
TSPO	Translocator Protein
MIR9-1	MicroRNA 9-1
MIR124-1	MicroRNA 124-1
MMP2	Matrix Metallopeptidase 2
F11	Coagulation Factor XI
MYBPC3	Myosin Binding Protein C3
CKM	Creatine Kinase, M-Type
ACTB	Actin Beta
GRN	Granulin Precursor
PCSK9	Proprotein Convertase Subtilisin/Kexin Type 9
SERPINA3	Serpin Family A Member 3
ATP1A3	ATPase Na+/K+ Transporting Subunit Alpha 3
CCM2	CCM2 Scaffold Protein
AVP	Arginine Vasopressin
PKD1	Polycystin 1, Transient Receptor Potential Channel Interacting
SCN8A	Sodium Voltage-Gated Channel Alpha Subunit 8
PRKG1	Protein Kinase CGMP-Dependent 1
MIR134	MicroRNA 134
MIR199A1	MicroRNA 199a-1
PIGQ	Phosphatidylinositol Glycan Anchor Biosynthesis Class Q
WDR37	WD Repeat Domain 37
GHRL	Ghrelin And Obestatin Prepropeptide
COL3A1	Collagen Type III Alpha 1 Chain
NGB	Neuroglobin
PTGIS	Prostaglandin I2 Synthase
MIR130A	MicroRNA 130a
LIPC	Lipase C, Hepatic Type
MIR221	MicroRNA 221
F2R	Coagulation Factor II Thrombin Receptor
IFNG	Interferon Gamma
MIR342	MicroRNA 342
MMP12	Matrix Metallopeptidase 12
TGFBR2	Transforming Growth Factor Beta Receptor 2
CYP11B2	Cytochrome P450 Family 11 Subfamily B Member 2
NPY	Neuropeptide Y
SLC6A4	Solute Carrier Family 6 Member 4
CCR5	C-C Motif Chemokine Receptor 5
LOC106627981	GBA Recombination Region
GNB3	G Protein Subunit Beta 3
LCN2	Lipocalin 2
TET2	Tet Methylcytosine Dioxygenase 2
UCP2	Uncoupling Protein 2
ALDH2	Aldehyde Dehydrogenase 2 Family Member
CASP1	Caspase 1
LINC01672	Long Intergenic Non-Protein Coding RNA 1672
DEPDC5	DEP Domain Containing 5, GATOR1 Subcomplex Subunit
PVALB	Parvalbumin
MIR133B	MicroRNA 133b
CD40LG	CD40 Ligand
UBE4A	Ubiquitination Factor E4A
TGFB2	Transforming Growth Factor Beta 2
NOTCH1	Notch Receptor 1
ABCB1	ATP Binding Cassette Subfamily B Member 1
MIR34A	MicroRNA 34a
GJA5	Gap Junction Protein Alpha 5
MIR210	MicroRNA 210
SMAD5-AS1	SMAD5 Antisense RNA 1
PROCR	Protein C Receptor

GATA4	GATA Binding Protein 4
ABCC9	ATP Binding Cassette Subfamily C Member 9
ANGPT2	Angiopoietin 2
GSR	Glutathione-Disulfide Reductase
HSPA1A	Heat Shock Protein Family A (Hsp70) Member 1A
SLC1A3	Solute Carrier Family 1 Member 3
CACNA1A	Calcium Voltage-Gated Channel Subunit Alpha1 A
PDE5A	Phosphodiesterase 5A
ABCC8	ATP Binding Cassette Subfamily C Member 8
HP	Haptoglobin
MBP	Myelin Basic Protein
LEP	Leptin
PGR-AS1	PGR Antisense RNA 1
JAG1	Jagged Canonical Notch Ligand 1
KDR	Kinase Insert Domain Receptor
SETD1B	SET Domain Containing 1B, Histone Lysine Methyltransferase
TGFB3	Transforming Growth Factor Beta 3
TNFRSF1A	TNF Receptor Superfamily Member 1A
EPHX2	Epoxide Hydrolase 2
MYH7	Myosin Heavy Chain 7
PWAR1	Prader Willi/Angelman Region RNA 1
TSHR	Thyroid Stimulating Hormone Receptor
APTX	Aprataxin
PDE3A	Phosphodiesterase 3A
ADRB1	Adrenoceptor Beta 1
MTOR	Mechanistic Target Of Rapamycin Kinase
F8	Coagulation Factor VIII
MIR499A	MicroRNA 499a
WWOX	WW Domain Containing Oxidoreductase
CD40	CD40 Molecule
CORIN	Corin, Serine Peptidase
HEY2	Hes Related Family BHLH Transcription Factor With YRPW Motif 2
FGG	Fibrinogen Gamma Chain
SLC1A2	Solute Carrier Family 1 Member 2
MB	Myoglobin
CDKN2B	Cyclin Dependent Kinase Inhibitor 2B
SMAD4	SMAD Family Member 4
ADORA1	Adenosine A1 Receptor
CDKN2A	Cyclin Dependent Kinase Inhibitor 2A
PTGS1	Prostaglandin-Endoperoxide Synthase 1
ARMS2	Age-Related Maculopathy Susceptibility 2
MYMY1	Moyamoya Disease 1
PIK3CG	Phosphatidylinositol-4,5-Bisphosphate 3-Kinase Catalytic Subunit Gamma
GP6	Glycoprotein VI Platelet
TGFBR1	Transforming Growth Factor Beta Receptor 1
APOA5	Apolipoprotein A5
CPA6	Carboxypeptidase A6
KCNT1	Potassium Sodium-Activated Channel Subfamily T Member 1
KMT2E	Lysine Methyltransferase 2E (Inactive)
NBEA	Neurobeachin
TTC21B	Tetratricopeptide Repeat Domain 21B
OTUD6B	OTU Deubiquitinase 6B
TANC2	Tetratricopeptide Repeat, Ankyrin Repeat And Coiled-Coil Containing 2
CEP128	Centrosomal Protein 128

Table S3. Ischemic stroke targets of RPR from OMIM databases.

Gene	name
ACE	Angiotensin I converting enzyme
ALOX5AP	Arachidonate 5-lipoxygenase-activating protein
GP1BA	Glycoprotein Ib
STRK1	Salt tolerance receptor-like cytoplasmic kinase 1

NOS3	Nitric Oxide Synthase 3
VWF	Von Willebrand factor
FBN1	Fibrillin 1
COL4A1	Collagen Type IV Alpha 1 Chain
TNF	Tumor necrosis factor
FGB	Fibrinogen Beta Chain
IL6	Interleukin 6
ACSL4	Acyl-CoA Synthetase Long Chain Family Member 4
HIF1A	Hypoxia inducible factor 1 subunit alpha
SERPINC1	Serpin Family C Member 1
JAK2	Janus Kinase 2
COX2	Cytochrome c oxidase subunit II

Table S4. Ischemic stroke targets of RPR from DisGeNET databases.

Gene	name
NOS3	Nitric oxide synthase 3
NOTCH3	Notch receptor 3
ACE	Angiotensin I converting enzyme
COX2	Cytochrome c oxidase subunit II
PRKCH	Protein kinase C eta
FBN1	Fibrillin 1
GRN	Granulin precursor
PLAT	Plasminogen activator, tissue type
ACSL4	Acyl-CoA synthetase long chain family member 4
CRP	C-reactive protein
VWF	Von Willebrand factor
CYTB	Cytochrome b
HBB	Hemoglobin subunit beta
F2	Coagulation factor II, thrombin
MTHFR	Methylenetetrahydrofolate reductase
AVP	Arginine vasopressin
GFAP	Glial fibrillary acidic protein
IGF1	Insulin like growth factor 1
IL10	Interleukin 10
MMP2	Matrix metallopeptidase 2
SELP	Selectin P
CCL2	C-C motif chemokine ligand 2
EEF1A2	Eukaryotic translation elongation factor 1 alpha 2
TNF	Tumor necrosis factor
ICAM1	Intercellular adhesion molecule 1
VEGFA	Vascular endothelial growth factor A
HBA2	Hemoglobin subunit alpha 2
IL6	Interleukin 6
HIF1A	Hypoxia inducible factor 1 subunit alpha
TLR4	Toll like receptor 4

Table S5. Total ischemic stroke targets of RPR.

Gene	name
NOTCH3	Notch Receptor 3
F5	Coagulation Factor V
ACE	Angiotensin I Converting Enzyme
F2	Coagulation Factor II, Thrombin
NOS3	Nitric Oxide Synthase 3
MTHFR	Methylenetetrahydrofolate Reductase
MT-TL1	Mitochondrially Encoded TRNA-Leu (UUA/G) 1
ALOX5AP	Arachidonate 5-Lipoxygenase Activating Protein
GP1BA	Glycoprotein Ib Platelet Subunit Alpha
PRKCH	Protein Kinase C Eta
RNF213	Ring Finger Protein 213
FBN1	Fibrillin 1

COL4A1	Collagen Type IV Alpha 1 Chain
BDNF-AS	BDNF Antisense RNA
CRP	C-Reactive Protein
FGB	Fibrinogen Beta Chain
APOE	Apolipoprotein E
IL6	Interleukin 6
ADA2	Adenosine Deaminase 2
APOB	Apolipoprotein B
MALAT1	Metastasis Associated Lung Adenocarcinoma Transcript 1
MIAT	Myocardial Infarction Associated Transcript
TNF	Tumor Necrosis Factor
MT-CYB	Mitochondrially Encoded Cytochrome B
MT-ND1	Mitochondrially Encoded NADH:Ubiquinone Oxidoreductase Core Subunit 1
LDLR	Low Density Lipoprotein Receptor
LOC132090228	Neanderthal Introgressed Variant-Containing Enhancer Experimental_34691
MT-TK	Mitochondrially Encoded TRNA-Lys (AAA/G)
SERPINE1	Serpin Family E Member 1
PLAT	Plasminogen Activator, Tissue Type
MT-TS1	Mitochondrially Encoded TRNA-Ser (UCN) 1
CDKN2B-AS1	CDKN2B Antisense RNA 1
MT-ND5	Mitochondrially Encoded NADH:Ubiquinone Oxidoreductase Core Subunit 5
MEG3	Maternally Expressed 3
HTRA1	HtrA Serine Peptidase 1
APP	Amyloid Beta Precursor Protein
ACSL4	Acyl-CoA Synthetase Long Chain Family Member 4
PDE4D	Phosphodiesterase 4D
MT-CO3	Mitochondrially Encoded Cytochrome C Oxidase III
PIK3CA	Phosphatidylinositol-4,5-Bisphosphate 3-Kinase Catalytic Subunit Alpha
CBS	Cystathionine Beta-Synthase
COL4A2	Collagen Type IV Alpha 2 Chain
POLG	DNA Polymerase Gamma, Catalytic Subunit
ACTA2	Actin Alpha 2, Smooth Muscle
MT-TI	Mitochondrially Encoded TRNA-Ile (AUU/C)
CD36	CD36 Molecule (CD36 Blood Group)
GLA	Galactosidase Alpha
BDNF	Brain Derived Neurotrophic Factor
MT-TL2	Mitochondrially Encoded TRNA-Leu (CUN) 2
MMP9	Matrix Metallopeptidase 9
IL1B	Interleukin 1 Beta
MT-CO1	Mitochondrially Encoded Cytochrome C Oxidase I
POLGARF	POLG Alternative Reading Frame
VWF	Von Willebrand Factor
MT-TP	Mitochondrially Encoded TRNA-Pro (CCN)
NPPA	Natriuretic Peptide A
SERPINC1	Serpin Family C Member 1
AGT	Angiotensinogen
MT-ND6	Mitochondrially Encoded NADH:Ubiquinone Oxidoreductase Core Subunit 6
SELP	Selectin P
NPPB	Natriuretic Peptide B
H19	H19 Imprinted Maternally Expressed Transcript
MT-ND4	Mitochondrially Encoded NADH:Ubiquinone Oxidoreductase Core Subunit 4
FGA	Fibrinogen Alpha Chain
IL10	Interleukin 10
TREX1	Three Prime Repair Exonuclease 1
MT-ATP6	Mitochondrially Encoded ATP Synthase Membrane Subunit 6
PON1	Paraoxonase 1
MT-CO2	Mitochondrially Encoded Cytochrome C Oxidase II
JAK2	Janus Kinase 2
APOH	Apolipoprotein H
LPA	Lipoprotein(A)
SELE	Selectin E
INS	Insulin

EDN1	Endothelin 1
F3	Coagulation Factor III, Tissue Factor
AGTR1	Angiotensin II Receptor Type 1
TLR4	Toll Like Receptor 4
ENG	Endoglin
ITGB3	Integrin Subunit Beta 3
ATRIP	ATR Interacting Protein
ATRIP-TREX1	ATRIP-TREX1 Readthrough
ALB	Albumin
CYP2C19	Cytochrome P450 Family 2 Subfamily C Member 19
HIF1A	Hypoxia Inducible Factor 1 Subunit Alpha
TP53	Tumor Protein P53
MTR	5-Methyltetrahydrofolate-Homocysteine Methyltransferase
PLA2G7	Phospholipase A2 Group VII
PPARG	Peroxisome Proliferator Activated Receptor Gamma
ICAM1	Intercellular Adhesion Molecule 1
HOTAIR	HOX Transcript Antisense RNA
THBD	Thrombomodulin
CST3	Cystatin C
LPL	Lipoprotein Lipase
ENO2	Enolase 2
GAS5	Growth Arrest Specific 5
ADAMTS13	ADAM Metallopeptidase With Thrombospondin Type 1 Motif 13
RPL36A-HNRNPH2	RPL36A-HNRNPH2 Readthrough
GUCY1A1	Guanylate Cyclase 1 Soluble Subunit Alpha 1
SCN5A	Sodium Voltage-Gated Channel Alpha Subunit 5
MAPT	Microtubule Associated Protein Tau
PTGS2	Prostaglandin-Endoperoxide Synthase 2
REN	Renin
EPO	Erythropoietin
TGFB1	Transforming Growth Factor Beta 1
MIR126	MicroRNA 126
KAT6B	Lysine Acetyltransferase 6B
VEGFA	Vascular Endothelial Growth Factor A
CCL2	C-C Motif Chemokine Ligand 2
GFAP	Glial Fibrillary Acidic Protein
ADIPOQ	Adiponectin, C1Q And Collagen Domain Containing
F7	Coagulation Factor VII
VCAM1	Vascular Cell Adhesion Molecule 1
LMNA	Lamin A/C
S100B	S100 Calcium Binding Protein B
MIR21	MicroRNA 21
APOA1	Apolipoprotein A1
ITGA2	Integrin Subunit Alpha 2
P2RY12	Purinergic Receptor P2Y12
MIR155	MicroRNA 155
IL1A	Interleukin 1 Alpha
DIAPH1	Diaphanous Related Formin 1
F13A1	Coagulation Factor XIII A Chain
ABCC6	ATP Binding Cassette Subfamily C Member 6
F12	Coagulation Factor XII
GRIN2B	Glutamate Ionotropic Receptor NMDA Type Subunit 2B
HBB	Hemoglobin Subunit Beta
MYH11	Myosin Heavy Chain 11
NLRP3	NLR Family Pyrin Domain Containing 3
IL18	Interleukin 18
TNNI3	Troponin I3, Cardiac Type
LOX	Lysyl Oxidase
SLC2A1	Solute Carrier Family 2 Member 1
SOD2-OT1	SOD2 Overlapping Transcript 1
MIR125A	MicroRNA 125a
HMGB1	High Mobility Group Box 1

MIR140	MicroRNA 140
PECAM1	Platelet And Endothelial Cell Adhesion Molecule 1
TNNT2	Troponin T2, Cardiac Type
PF4	Platelet Factor 4
RNF213-AS1	RNF213 Antisense RNA 1
MIR223	MicroRNA 223
MPO	Myeloperoxidase
VKORC1	Vitamin K Epoxide Reductase Complex Subunit 1
IL1RN	Interleukin 1 Receptor Antagonist
CERNA3	Competing Endogenous LncRNA 3 For MiR-645
MIR144	MicroRNA 144
MIR146A	MicroRNA 146a
ELN	Elastin
TTR	Transthyretin
PSEN1	Presenilin 1
CASP3	Caspase 3
SOD1	Superoxide Dismutase 1
CXCL12	C-X-C Motif Chemokine Ligand 12
KCNJ5	Potassium Inwardly Rectifying Channel Subfamily J Member 5
MYLK	Myosin Light Chain Kinase
PITX2	Paired Like Homeodomain 2
CXCL8	C-X-C Motif Chemokine Ligand 8
PLG	Plasminogen
HMOX1	Heme Oxygenase 1
ATP1A2	ATPase Na+/K+ Transporting Subunit Alpha 2
FGF2	Fibroblast Growth Factor 2
PRNP	Prion Protein (Kanno Blood Group)
EDNRA	Endothelin Receptor Type A
CREB1	CAMP Responsive Element Binding Protein 1
IL4	Interleukin 4
MAP2	Microtubule Associated Protein 2
KCNQ1	Potassium Voltage-Gated Channel Subfamily Q Member 1
NGF	Nerve Growth Factor
IGF1	Insulin Like Growth Factor 1
HSPA4	Heat Shock Protein Family A (Hsp70) Member 4
ESR1	Estrogen Receptor 1
GBA1	Glucosylceramidase Beta 1
PROZ	Protein Z, Vitamin K Dependent Plasma Glycoprotein
CETP	Cholesteryl Ester Transfer Protein
ACE2	Angiotensin Converting Enzyme 2
PON2	Paraoxonase 2
PIK3C2A	Phosphatidylinositol-4-Phosphate 3-Kinase Catalytic Subunit Type 2 Alpha
CKB	Creatine Kinase B
LTA	Lymphotoxin Alpha
BCL2	BCL2 Apoptosis Regulator
KNG1	Kininogen 1
SCN1A	Sodium Voltage-Gated Channel Alpha Subunit 1
THPO	Thrombopoietin
CYCS	Cytochrome C, Somatic
GRIN2A	Glutamate Ionotropic Receptor NMDA Type Subunit 2A
MMP3	Matrix Metallopeptidase 3
NR4A2	Nuclear Receptor Subfamily 4 Group A Member 2
GPT	Glutamic--Pyruvic Transaminase
RETN	Resistin
MECP2	Methyl-CpG Binding Protein 2
TTN	Titin
MIR17	MicroRNA 17
TUG1	Taurine Up-Regulated 1
SH2B3	SH2B Adaptor Protein 3
HMGCR	3-Hydroxy-3-Methylglutaryl-CoA Reductase
HDAC9	Histone Deacetylase 9
CYP2C9	Cytochrome P450 Family 2 Subfamily C Member 9

KRIT1	KRIT1 Ankyrin Repeat Containing
ENPP1	Ectonucleotide Pyrophosphatase/Phosphodiesterase 1
AKT1	AKT Serine/Threonine Kinase 1
F10	Coagulation Factor X
MIR143	MicroRNA 143
NES	Nestin
CALR	Calreticulin
CD14	CD14 Molecule
FLNA	Filamin A
DARS2	Aspartyl-TRNA Synthetase 2, Mitochondrial
CTSA	Cathepsin A
SOD2	Superoxide Dismutase 2
NOS1	Nitric Oxide Synthase 1
SULT1A3	Sulfotransferase Family 1A Member 3
ABCA1	ATP Binding Cassette Subfamily A Member 1
MIR150	MicroRNA 150
ADRB2	Adrenoceptor Beta 2
MIR145	MicroRNA 145
ANGPT1	Angiopoietin 1
MIR29A	MicroRNA 29a
GPX3	Glutathione Peroxidase 3
ACVRL1	Activin A Receptor Like Type 1
OLR1	Oxidized Low Density Lipoprotein Receptor 1
APOC3	Apolipoprotein C3
GDNF	Glial Cell Derived Neurotrophic Factor
TNFRSF11B	TNF Receptor Superfamily Member 11b
ALOX5	Arachidonate 5-Lipoxygenase
MIR146B	MicroRNA 146b
ADD1	Adducin 1
TMX2-CTNND1	TMX2-CTNND1 Readthrough (NMD Candidate)
PPBP	Pro-Platelet Basic Protein
SLC2A10	Solute Carrier Family 2 Member 10
ADM	Adrenomedullin
MIR142	MicroRNA 142
NKX2-5	NK2 Homeobox 5
NFE2L2	NFE2 Like BZIP Transcription Factor 2
SMAD3	SMAD Family Member 3
SPP1	Secreted Phosphoprotein 1
STAT3	Signal Transducer And Activator Of Transcription 3
MPL	MPL Proto-Oncogene, Thrombopoietin Receptor
ZFHX3	Zinc Finger Homeobox 3
ITGA2B	Integrin Subunit Alpha 2b
SIRT1	Sirtuin 1
MIR98	MicroRNA 98
TSPO	Translocator Protein
MIR9-1	MicroRNA 9-1
MIR124-1	MicroRNA 124-1
MMP2	Matrix Metallopeptidase 2
F11	Coagulation Factor XI
MYBPC3	Myosin Binding Protein C3
CKM	Creatine Kinase, M-Type
ACTB	Actin Beta
GRN	Granulin Precursor
PCSK9	Proprotein Convertase Subtilisin/Kexin Type 9
SERPINA3	Serpin Family A Member 3
ATP1A3	ATPase Na+/K+ Transporting Subunit Alpha 3
CCM2	CCM2 Scaffold Protein
AVP	Arginine Vasopressin
PKD1	Polycystin 1, Transient Receptor Potential Channel Interacting
SCN8A	Sodium Voltage-Gated Channel Alpha Subunit 8
PRKG1	Protein Kinase CGMP-Dependent 1
MIR134	MicroRNA 134

MIR199A1	MicroRNA 199a-1
PIGQ	Phosphatidylinositol Glycan Anchor Biosynthesis Class Q
WDR37	WD Repeat Domain 37
GHRL	Ghrelin And Obestatin Prepropeptide
COL3A1	Collagen Type III Alpha 1 Chain
NGB	Neuroglobin
PTGIS	Prostaglandin I2 Synthase
MIR130A	MicroRNA 130a
LIPC	Lipase C, Hepatic Type
MIR221	MicroRNA 221
F2R	Coagulation Factor II Thrombin Receptor
IFNG	Interferon Gamma
MIR342	MicroRNA 342
MMP12	Matrix Metallopeptidase 12
TGFBR2	Transforming Growth Factor Beta Receptor 2
CYP11B2	Cytochrome P450 Family 11 Subfamily B Member 2
NPY	Neuropeptide Y
SLC6A4	Solute Carrier Family 6 Member 4
CCR5	C-C Motif Chemokine Receptor 5
LOC106627981	GBA Recombination Region
GNB3	G Protein Subunit Beta 3
LCN2	Lipocalin 2
TET2	Tet Methylcytosine Dioxygenase 2
UCP2	Uncoupling Protein 2
ALDH2	Aldehyde Dehydrogenase 2 Family Member
CASP1	Caspase 1
LINC01672	Long Intergenic Non-Protein Coding RNA 1672
DEPDC5	DEP Domain Containing 5, GATOR1 Subcomplex Subunit
PVALB	Parvalbumin
MIR133B	MicroRNA 133b
CD40LG	CD40 Ligand
UBE4A	Ubiquitination Factor E4A
TGFB2	Transforming Growth Factor Beta 2
NOTCH1	Notch Receptor 1
ABCB1	ATP Binding Cassette Subfamily B Member 1
MIR34A	MicroRNA 34a
GJA5	Gap Junction Protein Alpha 5
MIR210	MicroRNA 210
SMAD5-AS1	SMAD5 Antisense RNA 1
PROCR	Protein C Receptor
GATA4	GATA Binding Protein 4
