# Supplementary figures and images for: Investigation into the Potential Mechanism of Radix Paeoniae Rubra Against Ischemic Stroke Based on Network Pharmacology
Source: Nutrients. 2024 Dec 23;16(24):4409. doi: 10.3390/nu16244409 (PMC11678013; doi:10.3390/nu16244409)

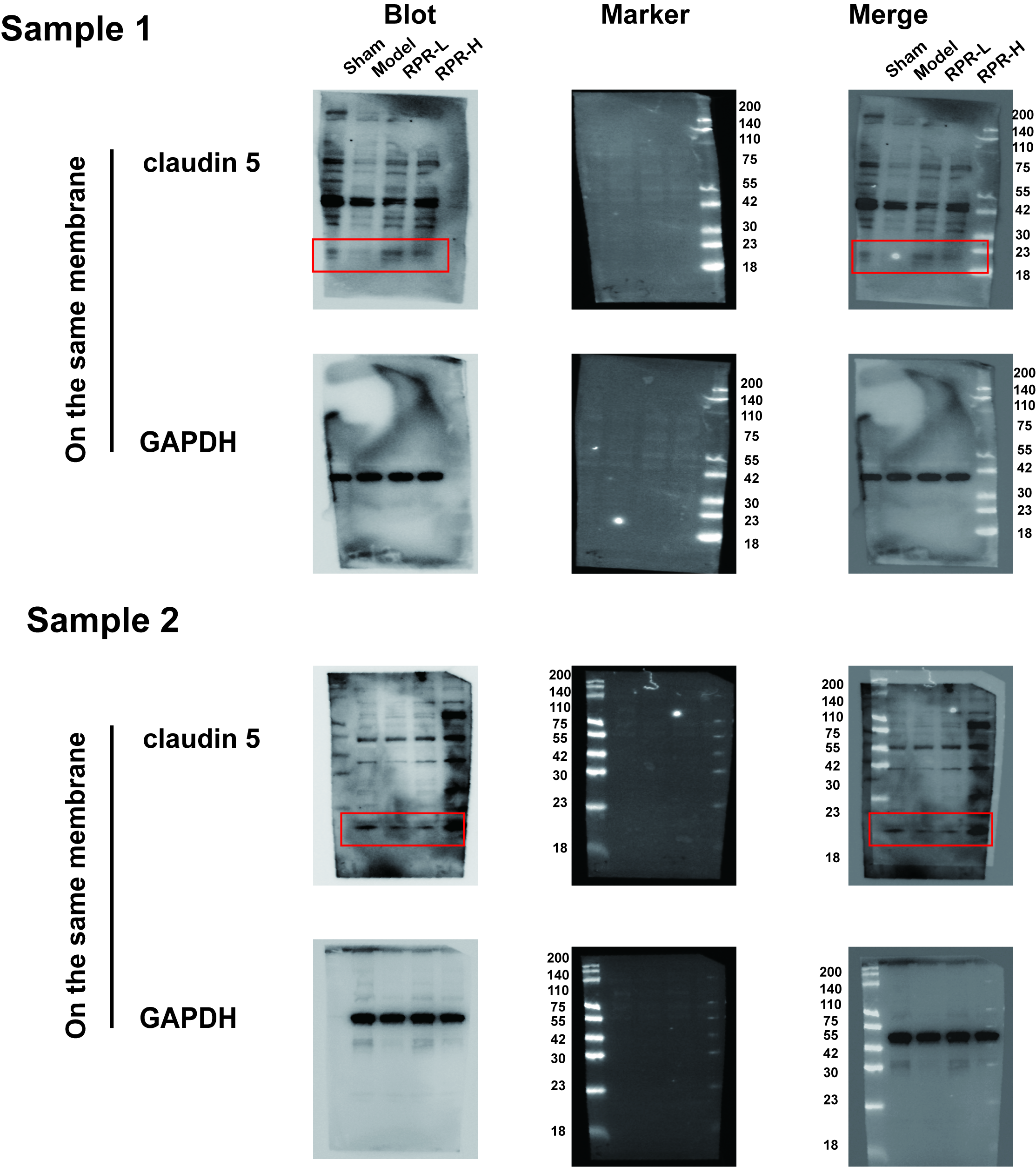

Supplement: Supplementary file 1 [file nutrients-16-04409-s001.zip › Supplementary file/Figure 4E/claudin 5.tif]

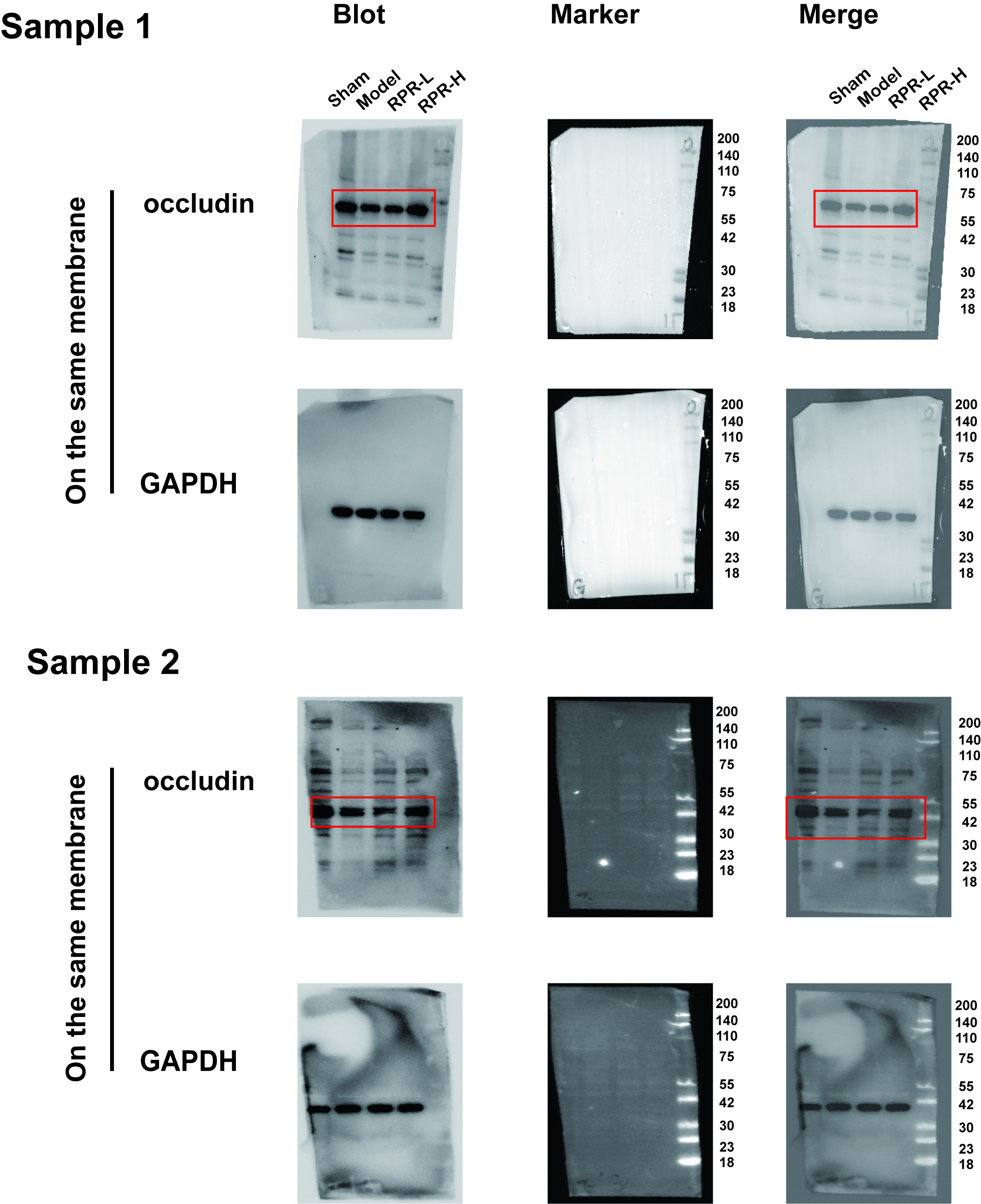

Supplement: Supplementary file 1 [file nutrients-16-04409-s001.zip › Supplementary file/Figure 4E/occludin.tif]

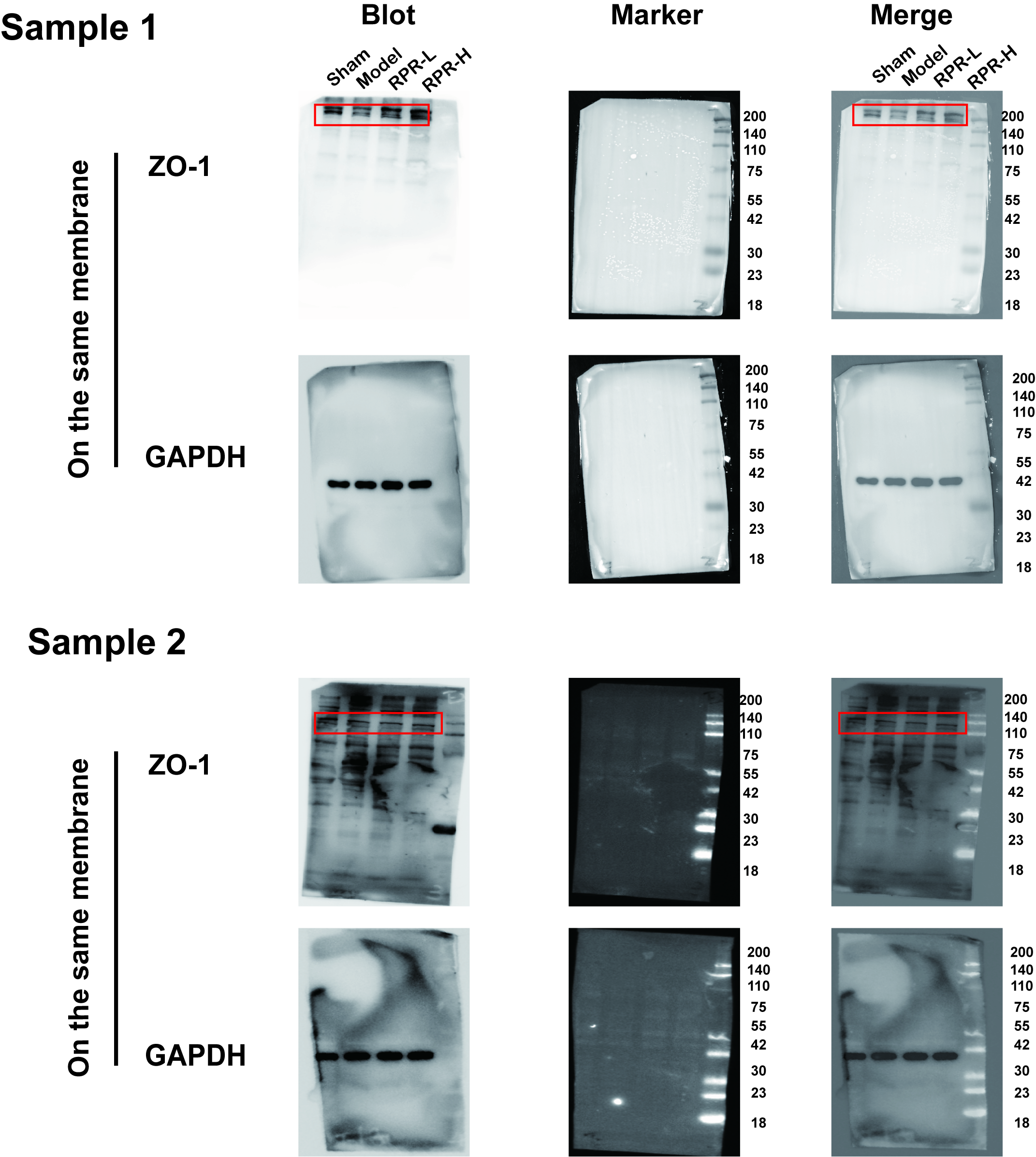

Supplement: Supplementary file 1 [file nutrients-16-04409-s001.zip › Supplementary file/Figure 4E/zo-1.tif]

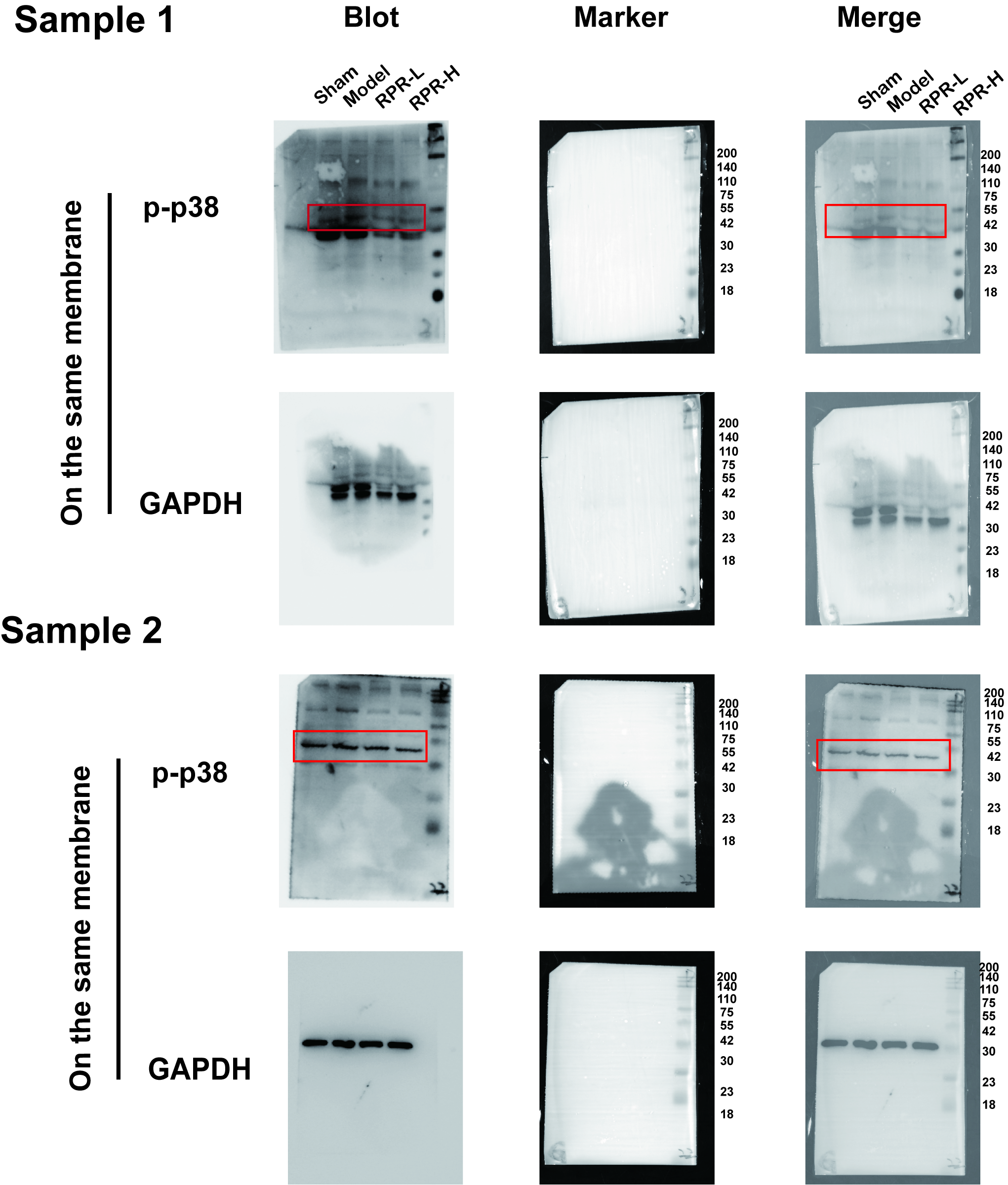

Supplement: Supplementary file 1 [file nutrients-16-04409-s001.zip › Supplementary file/Figure 5/Figure 5A/p-p38.tif]

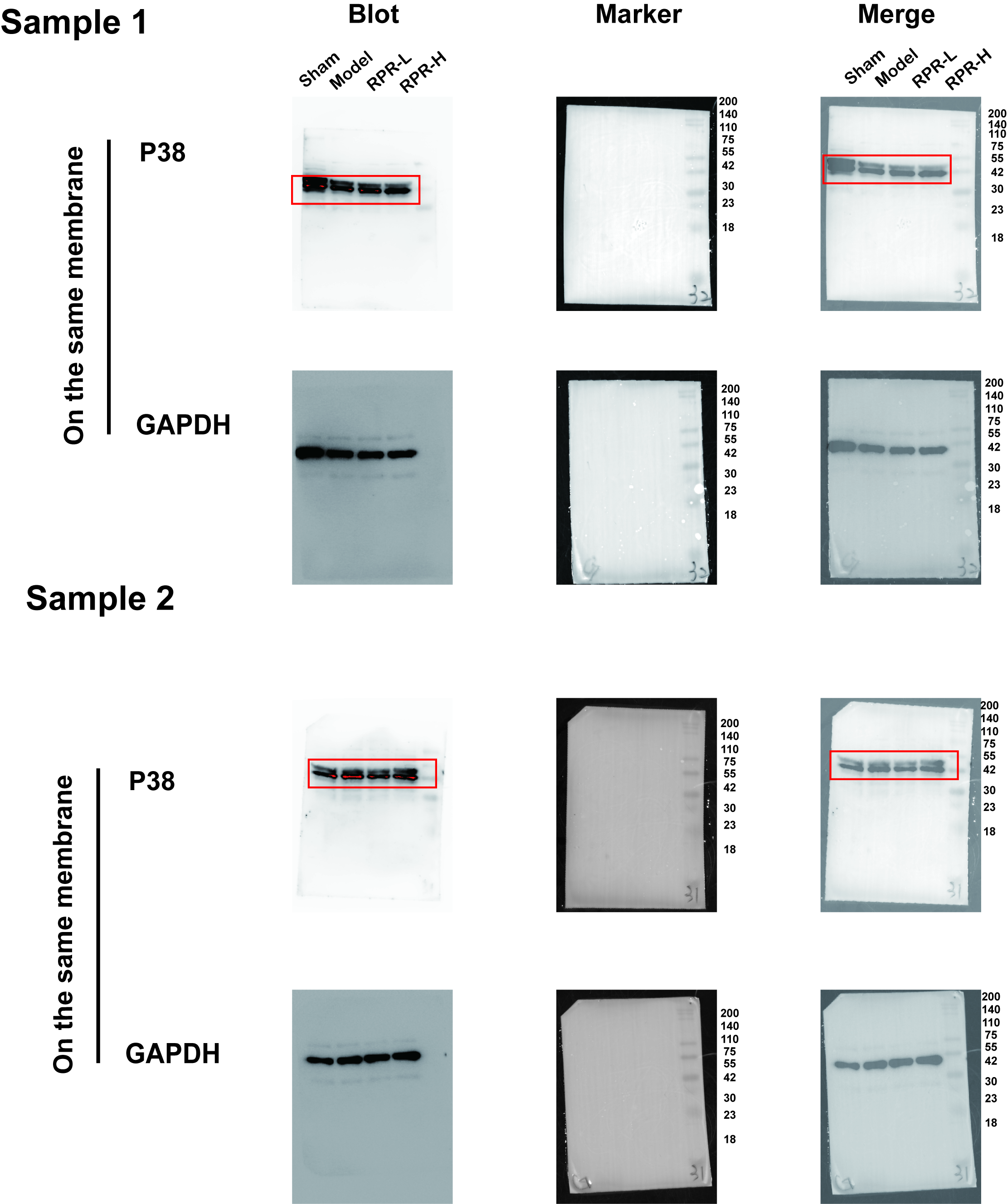

Supplement: Supplementary file 1 [file nutrients-16-04409-s001.zip › Supplementary file/Figure 5/Figure 5A/p38.tif]

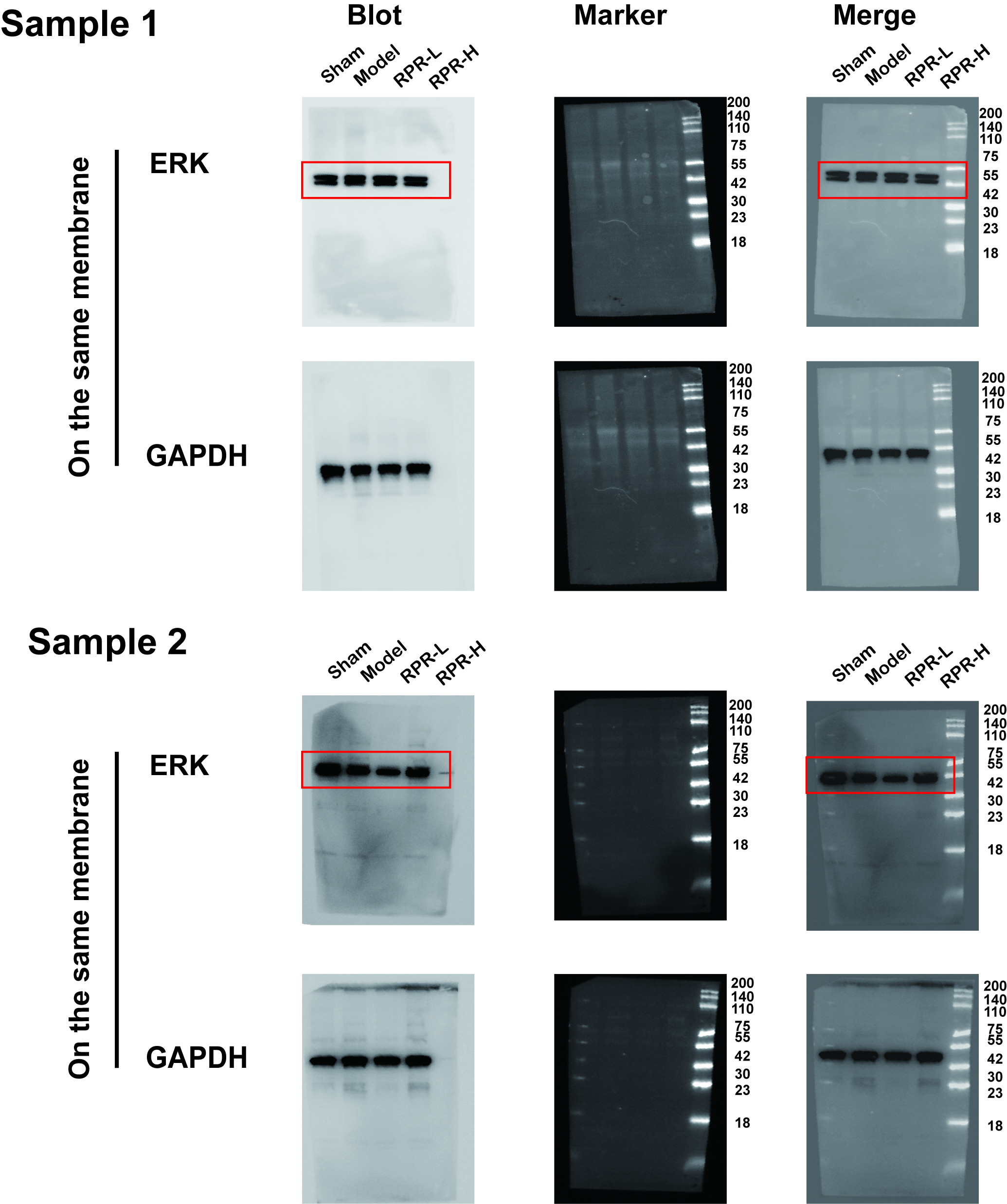

Supplement: Supplementary file 1 [file nutrients-16-04409-s001.zip › Supplementary file/Figure 5/Figure 5B/ERK.tif]

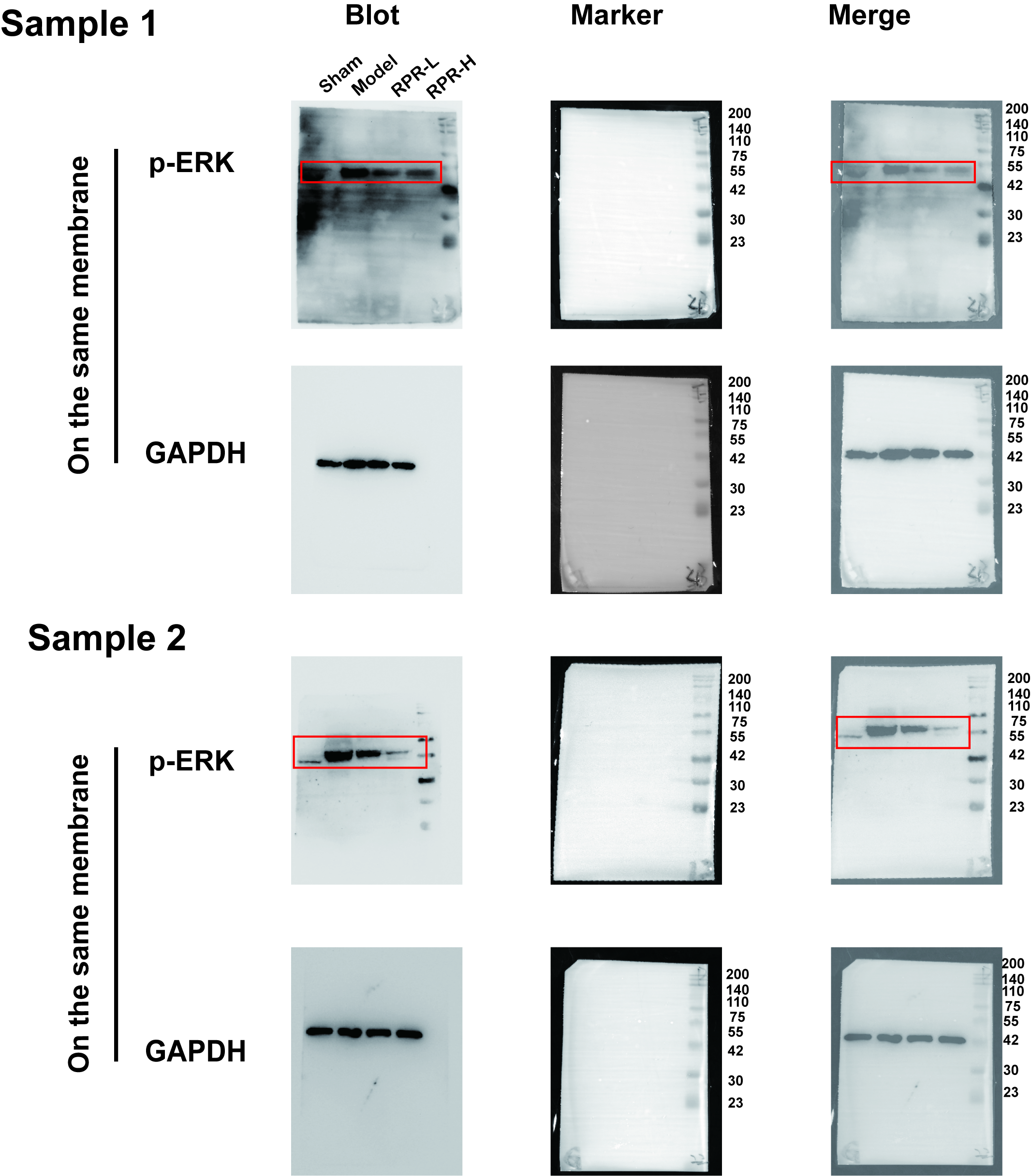

Supplement: Supplementary file 1 [file nutrients-16-04409-s001.zip › Supplementary file/Figure 5/Figure 5B/p-ERK.tif]

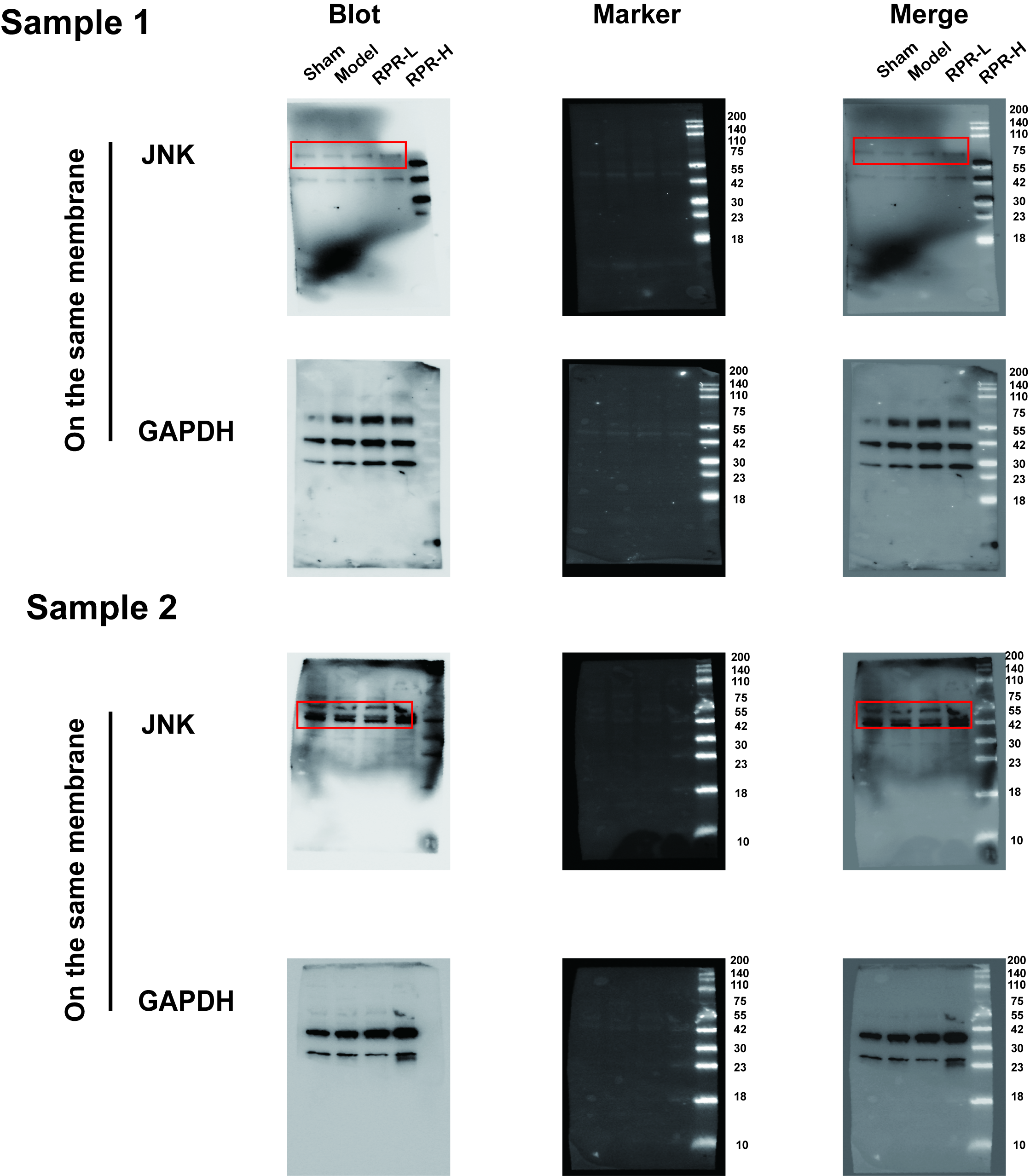

Supplement: Supplementary file 1 [file nutrients-16-04409-s001.zip › Supplementary file/Figure 5/Figure 5C/JNK.tif]

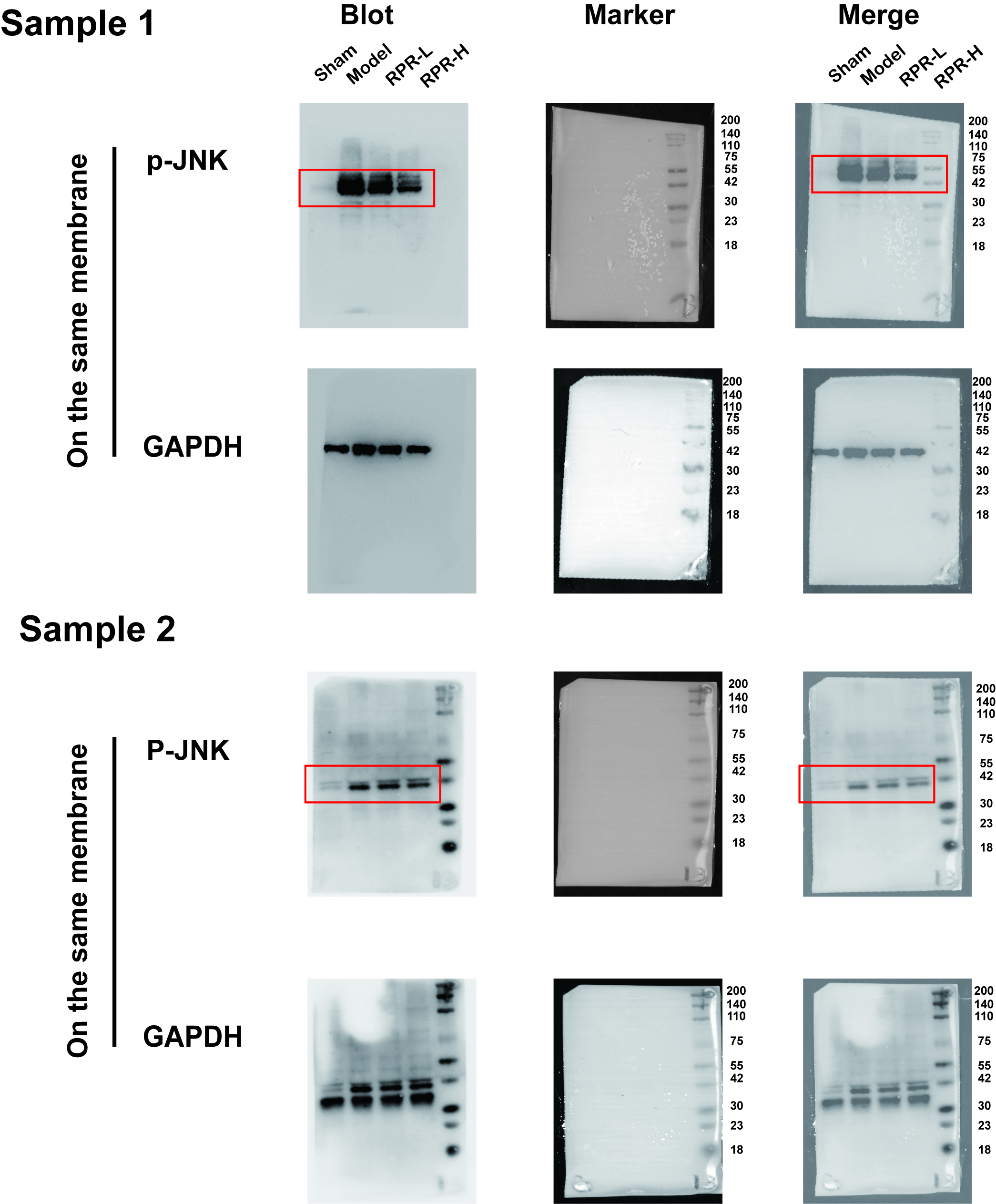

Supplement: Supplementary file 1 [file nutrients-16-04409-s001.zip › Supplementary file/Figure 5/Figure 5C/p-JNK.tif]

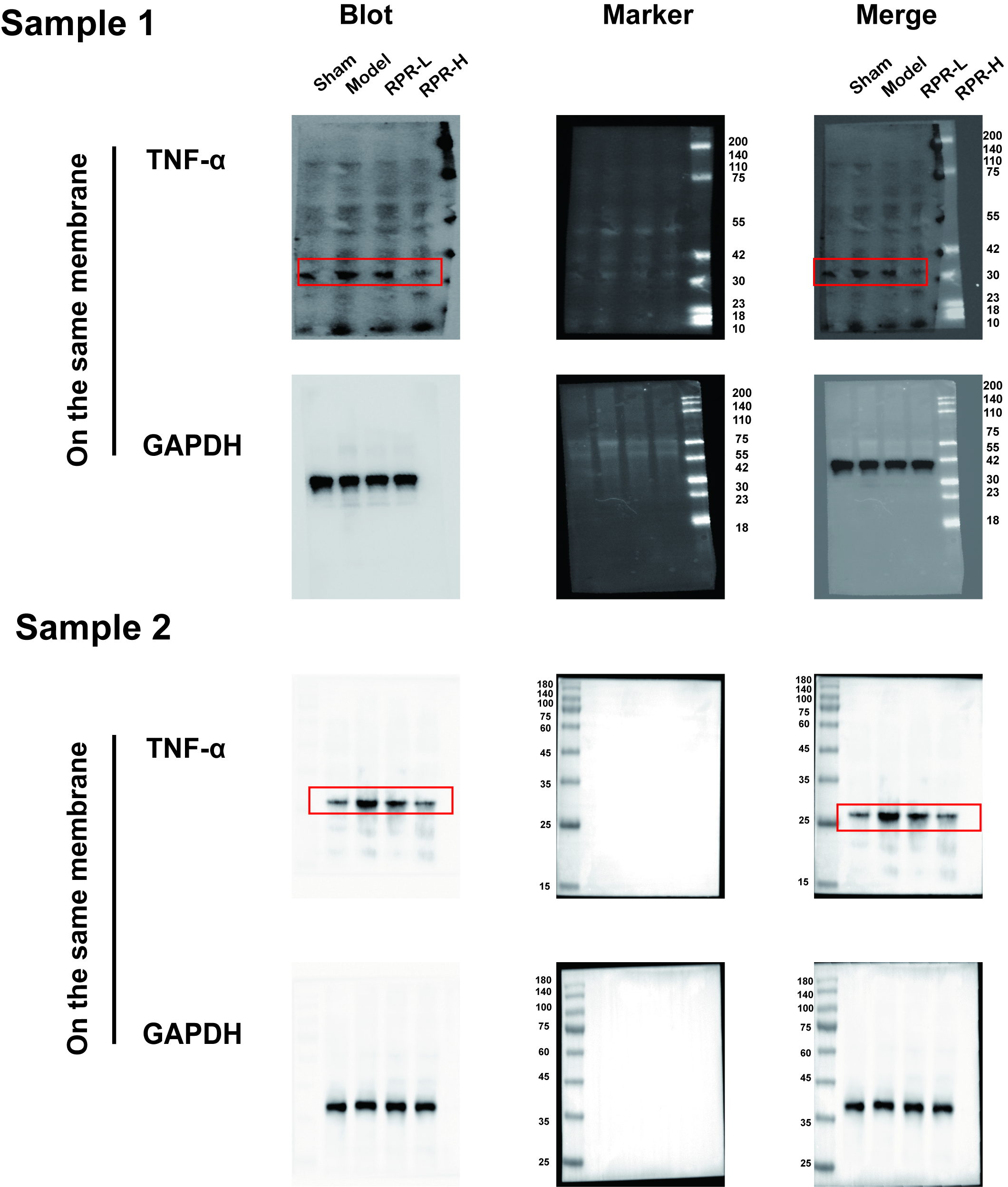

Supplement: Supplementary file 1 [file nutrients-16-04409-s001.zip › Supplementary file/Figure 5/Figure 5D/TNF.tif]
